# Development and validation of influenza forecasting for 64 temperate and tropical countries

**DOI:** 10.1371/journal.pcbi.1006742

**Published:** 2019-02-27

**Authors:** Sarah C. Kramer, Jeffrey Shaman

**Affiliations:** Department of Environmental Health Sciences, Mailman School of Public Health, Columbia University, New York, New York, United States of America; London School of Hygiene & Tropical Medicine, UNITED KINGDOM

## Abstract

Accurate forecasts of influenza incidence can be used to inform medical and public health decision-making and response efforts. However, forecasting systems are uncommon in most countries, with a few notable exceptions. Here we use publicly available data from the World Health Organization to generate retrospective forecasts of influenza peak timing and peak intensity for 64 countries, including 18 tropical and subtropical countries. We find that accurate and well-calibrated forecasts can be generated for countries in temperate regions, with peak timing and intensity accuracy exceeding 50% at four and two weeks prior to the predicted epidemic peak, respectively. Forecasts are significantly less accurate in the tropics and subtropics for both peak timing and intensity. This work indicates that, in temperate regions around the world, forecasts can be generated with sufficient lead time to prepare for upcoming outbreak peak incidence.

## Author summary

Influenza is responsible for an estimated 3–5 million cases and 300–650,000 deaths each year worldwide. If produced early enough, accurate forecasts of influenza activity could guide public health practitioners and medical professionals in preparing for an outbreak, reducing the subsequent morbidity and mortality. For example, hospitals could use these forecasts to determine how many beds will be needed when an outbreak is most intense. Despite this potential impact, influenza forecasts are primarily generated for the United States, with forecasts for other countries being comparatively rare. Here, we use publically available influenza data to forecast influenza activity in 64 countries. We find that accurate forecasts can be produced several weeks before the outbreak’s peak in temperate countries, where influenza outbreaks occur regularly during the winter. Forecast accuracy is lower in the tropics and subtropics, where outbreaks occur more sporadically. Overall, our results suggest that forecasts have potential as an important public health tool in many countries, not only in the US.

## Introduction

Forecasting is an important tool in a number of fields, including weather and climate [1–3], agriculture [4,5], air quality [6,7], and consumer activity [8–10]. When operationalized for use in real time, predictions from probabilistic forecasts can be used in decision-making to inform, for example, emergency food aid allocation [4] or profit maximization [8]. Recently, forecasting systems have also been developed for a range of infectious diseases of high public health concern, including influenza [11–20], norovirus [21], dengue [22–25], Ebola [26–29], and, most recently, Zika [30,31].

The ability to generate accurate, real-time forecasts of infectious disease activity has important implications for public health. Currently, response to infectious disease outbreaks is primarily reactive: medical and public health professionals attempt to deal with unexpected spikes of disease incidence as they occur. By providing information on when an outbreak is expected to peak and how many cases are expected at that peak, forecasts have the potential to create a paradigm shift in infectious disease control and public health decision-making. For example, hospitals expecting a patient surge might ensure that adequate resources are available, avoiding bed and staff shortages.

Seasonal influenza produces annual wintertime outbreaks in temperate regions, as well as sporadic outbreaks throughout the year in the tropics and subtropics [32,33]. The World Health Organization (WHO) estimates that influenza causes about 300,000–650,000 deaths and 3–5 million cases of severe illness each year [34]. To date, forecasts of influenza activity in the United States have been generated and operationalized [11,15]. However, while influenza forecasts have been generated for countries outside the US [13,14,17,18,35,36], these efforts are less numerous, and many countries have been ignored entirely. The tropics and subtropics are particularly neglected, with forecasts attempted for only Hong Kong [18] and Singapore [17]. This is true despite evidence suggesting that influenza burden in the tropics is similar to that in temperate regions [33].

The WHO collects influenza data year-round from several member states around the world. To our knowledge, no influenza forecasts have yet been generated using these data. Given differences in data collection procedures by country, and the importance of high data quality for generating accurate forecasts, whether these data can be used to generate accurate forecasts remains an open question. Here, we explore the following research questions: 1) Can the WHO data be used to generate accurate and well-calibrated retrospective forecasts at the country level?; 2) Does forecast accuracy significantly differ between temperate and tropical regions?; and 3) What factors are associated with substantial changes in forecast accuracy within both temperate and tropical regions? Based on past work, we expect that forecasting will be feasible in all regions, but that forecast accuracy will be substantially higher in temperate regions.

## Materials and methods

### Influenza data

**Fig 1:**
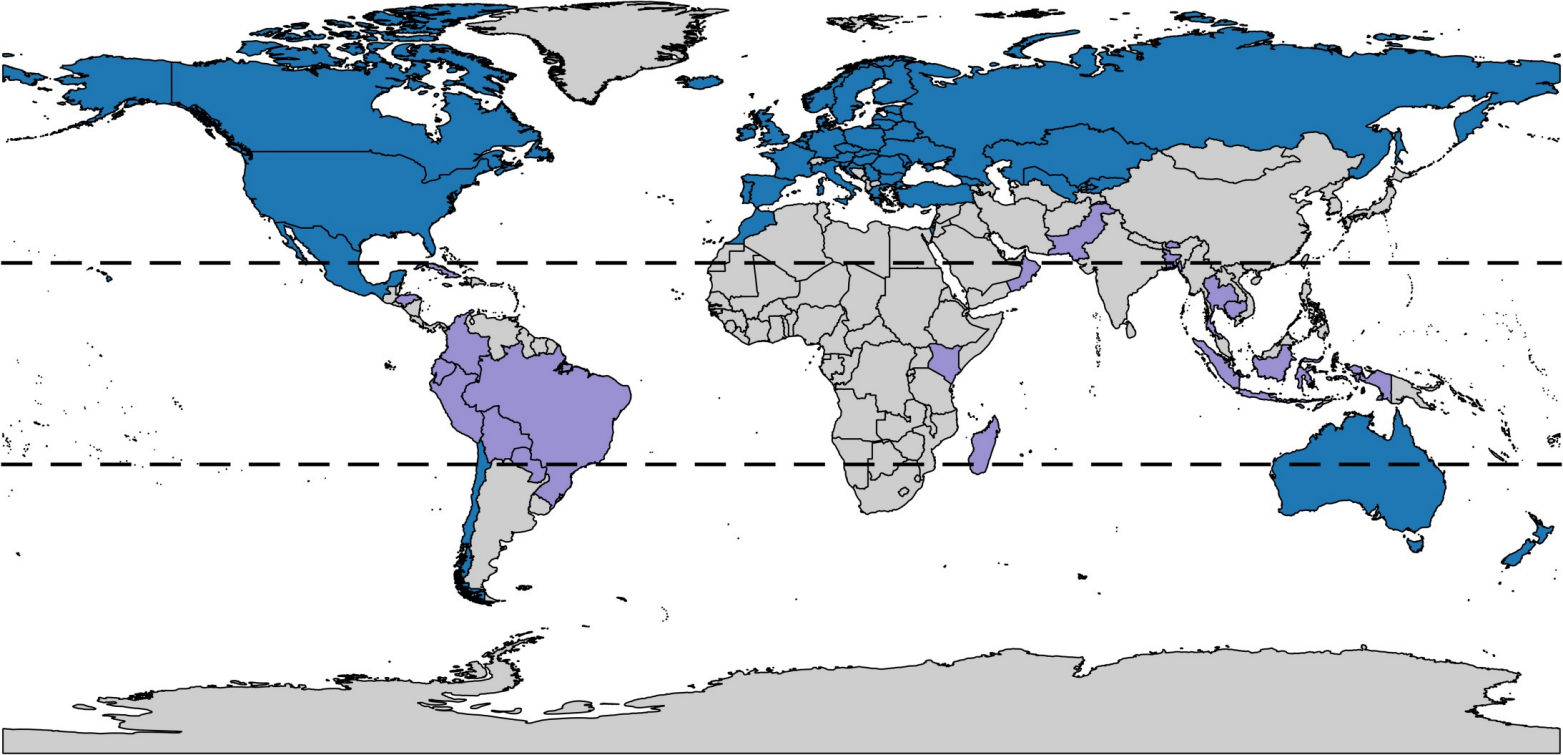
Countries with good quality syndromic and virologic data for at least one season. The black dotted lines demarcate the boundaries of the tropics. Countries classified as temperate are shaded in blue, and countries classified as tropical are shaded in purple. Of 64 countries, 18 were classified as tropical, and 3 as southern temperate.

Influenza syndromic and virologic data were obtained from WHO’s FluID [37] and FluNet [38] web-tools, respectively. Briefly, these systems contain aggregated influenza data from WHO member states, which are either submitted by member states directly or downloaded by the WHO from existing regional databases. Good quality (see S1 Text) syndromic and virologic data were available for at least one season from 64 countries, primarily in Europe and North America (see Figs 1 and S1). Countries were classified as temperate or tropical based on both their latitude and whether they demonstrated seasonal or sporadic influenza dynamics (see S1 Text). Overall, eighteen countries were classified as tropical, and three (Australia, New Zealand, and Chile) were located in the southern temperate region.

FluID data include diagnostic counts of influenza-like illness (ILI), acute respiratory infection (ARI), severe acute respiratory infection (SARI), and pneumonia, with different countries preferentially reporting different data types (see S1 Text for additional information). Because these data contain no information on laboratory testing, counts include both patients infected with influenza and patients infected by other pathogens that lead to similar signs and symptoms. To adjust for this lack of specificity, we use FluNet data, which includes the total number of tests performed for influenza and the number positive for influenza. Specifically, we multiply the syndromic case counts from the FluID tool by the proportion of tests positive for influenza in that same country during a given week. This calculation eliminates out-of-season syndromic cases that are unlikely to be due to influenza. Further, as the model used in this study (described below) simulates the transmission of a single pathogen, the removal of incidence due to non-influenza illness increases agreement between model input (data) and output. We refer to the resulting measures as ILI+, ARI+, SARI+, or pneumonia+, or, more broadly, syndromic+.

For this study, we focused specifically on seasonal influenza outbreaks, and excluded the 2009 pandemic from the main analysis. While pandemic outbreaks often produce a strong incidence signal that is forecastable [17], they typically appear out-of-season in temperate regions. Seasonal influenza outbreaks, on the other hand, occur with enough frequency that, even in the tropics, where outbreak timing is less regular, future epidemics are almost certain to occur within the year. To maintain a consistent forecasting approach, we therefore focus on seasonal influenza. We present results from forecasting the 2009 pandemic alone, as well as associated methods, in S1 Text and S19 Fig.

In addition, individual seasons were removed from the final dataset if: a) five or more consecutive weeks of data were missing near the outbreak peak (n = 2); b) the season consisted of fewer than 5 non-NA and non-zero data points (n = 2); c) the total attack rate of the season was less than 5% that of the largest outbreak (in other words, if case counts were unrealistically low; n = 5); d) data collection began or ended at the outbreak peak (n = 4); or e) no consecutive weeks of data were available (in other words, data were only available every other week; n = 1). We also removed data from 2010–11 in Mexico due to the continued disruption of typical seasonal patterns by the 2009 pandemic. Individual data points were removed if they occurred outside of the influenza season (as defined below under “Delineation of Influenza Seasons”) and were greater than 50% of the maximum value for the country over all seasons (n = 1). In total, 15 individual seasons were removed from the dataset, and 64 countries remained. In temperate regions, data were available for between one and seven seasons for each country for a total of 289 seasons. A complete list of countries and seasons used for forecasting can be found in S1 and S2 Tables, and the cleaned influenza data are available as S1 Dataset. Note that, for the seasonal forecasts, we began fitting tropical data at week 40 of 2010.

### Humidity data

Data on absolute humidity were obtained from NASA’s Global Land Data Assimilation System (GLDAS), which uses both observed data and modeling techniques to produce high-resolution surface meteorological data [39]. Data were available every three hours at a spatial resolution of 1°x1° for the years 1989–2008. Data from each grid cell were aggregated to the daily level, and anomalous records were identified by visual inspection and removed. Then, climatologies for each grid cell were generated by averaging daily specific humidity on each of 365 days across twelve to twenty years, depending on the amount of anomalous data removed. Finally, climatologies were aggregated to the country level by averaging the climatologies for all grid cells lying within a country, weighted by the proportion of the grid cell situated within the country in question. A more detailed description of how the humidity data were processed can be found in the S1 Text, and the processed data are available as S2 Dataset.

### Delineation of influenza seasons

The influenza season in temperate regions of the northern hemisphere is modeled as beginning in week 40 and ending in week 20 of the following year [40]. We shift these values by one half-year for temperate regions of the southern hemisphere; thus, the influenza season begins in week 14 and continues until week 46.

For tropical regions, where consistent seasonality in influenza infection patterns is not observed, the above methods are not sufficient. Individual outbreaks are instead identified using methodology previously described in [18]. Briefly, outbreak onsets are defined as the first of three consecutive weeks where ILI+ rates exceeded the 33rd percentile of non-zero ILI+ values across all available data for a country. The end of an outbreak is defined as the first of two consecutive weeks below this threshold. To ensure that sporadic spikes in influenza are not counted, we remove any outbreaks where ILI+ counts never exceeded three times its respective onset threshold value.

### Retrospective forecast generation

Country-level retrospective forecasts are developed using a model-data assimilation system consisting of: (1) influenza observations, as described above, (2) a model of influenza transmission, and (3) a filter to assimilate observations and optimize model simulation and ensemble forecast. The final two components are described here. These components differed slightly for temperate and tropical regions, and are therefore described separately.

#### Temperate regions

SIRS Model: We model influenza transmission in temperate regions using a compartmental, humidity-forced Susceptible-Infected-Recovered-Susceptible (SIRS) model, in which members of the model population move through compartments according to the following equations:

$$\frac{dS}{dt}=\frac{N-S-I}{L}-\frac{\beta \left(t\right)IS}{N}-\alpha$$

$$\frac{dI}{dt}=\frac{\beta \left(t\right)IS}{N}-\frac{I}{D}+\alpha$$ (1)

where *N* is the total model population size, set arbitrarily to 100,000 for all countries; *S* and *I* are the total number of people susceptible and infected, respectively; *t* is time in days; *β(t)* is the transmission rate at time *t*; *D* is the mean infectious period; *L* is the average duration of immunity before recovery; and *α* represents the rate of influenza importation from outside the model population, here set to 0.1, or 1 case per 10 days [12]. The basic reproductive number (*R*_*0*_), a key parameter in infectious disease epidemiology representing the average number of secondary infections arising from a single primary infection in a fully susceptible population, is related to *β(t)* and *D* by the expression $R_{0}\left(t\right)\;=\;\beta \left(t\right)D$.

Daily specific humidity modulates *R*_*0*_*(t)* as follows:

$$R_{0}\left(t\right)=e^{-180q\left(t\right)+\text{ln}\left(R_{0_{max}}-R_{0_{min}}\right)}+R_{0_{min}}$$ (2)

where *R*_*0max*_ is the maximum daily basic reproductive number, *R*_*0min*_ is the minimum daily basic reproductive number, and *q(t)* is the specific humidity on day *t* [12]. We set *a* equal to -180, based on laboratory regression of influenza virus survival on specific humidity [41]. Absolute humidity has been shown to increase influenza survival and transmission in laboratory experiments [41], and model studies indicate that lower absolute humidity during the winter is a significant driver of influenza seasonality in temperate regions [42]. Similar models have been used to forecast influenza at the city and state level in the US [11,12,42], and previous work has shown that inclusion of absolute humidity forcing significantly improves forecast performance [43].

Data Assimilation Methods: The above model is fit to the syndromic+ data using the Ensemble Adjustment Kalman Filter (EAKF), a data assimilation method used in weather forecasting [44]. In practice, we randomly initialize an ensemble of simulations (see Forecast Generation below for details) that are then integrated forward per the model equations. At each observation the integration is halted and the ensemble observed state is updated using the EAKF algorithm and that observation, per Bayes Rule:

$$p\left(X_{t}\text{|}O_{1:t}\right)\propto p(X_{t}|O_{1:\left(t-1\right)})\cdot p(O_{t}|X_{t})$$ (3)

where $p(X_{t}|O_{1:\left(t-1\right)})$ is the prior distribution of the observed model state (here, the number of newly infected individuals) given all observations prior to time *t*, $p(O_{t}|X_{t})$ is the likelihood of the observation at time *t* given the model state at time *t*, and $p\left(X_{t}|O_{1:t}\right)$ is the posterior distribution of the model state given all observations up to and including time *t*. The probability of the model state is based on the distribution of the ensemble of simulations. Unobserved state variables and parameters (*S*, *R*_*0max*_, *R*_*0min*_, *D*, and *L*) are updated according to cross-ensemble covariability with the observed model state. More details on the EAKF’s implementation can be found in S1 Text, as well as in [12,19,44,45].

Forecast Generation: A forecast for week *t* is produced by first iteratively fitting the ensemble of simulations to local observations from the beginning of the season up to and including week *t*, and then integrating the ensemble until the end of the epidemic period using the final inferred states and parameters from the training period (i.e. the posterior at week *t*). This process is repeated throughout the season for weeks 44 through 69 in the northern hemisphere, and weeks 18 through 43 in the southern hemisphere. Thus each ensemble forecast assimilates 5 to 30 weeks of training data. Prior to simulation and forecast, initial values of states and parameters for each ensemble member are randomly selected using Latin hypercube sampling from ranges previously reported $(1.3\leq R_{0_{max}}\leq 4.0,$ $0.8\;\leq R_{0_{min}}\leq 1.2,$ 1.5≤D≤7.0, 1.5≤D≤7.0, 365≤L≤3650) [12]. In order to account for any stochastic effects during this initialization 5 separate 300-member ensembles were initialized and used to generate forecasts for each location and season. The average results over all ensembles are reported. Variance within an ensemble permits assessment of forecast uncertainty [12].

#### Tropical regions

For the most part, the procedure used to generate retrospective forecasts in the tropics is similar to that used in temperate zones. Differences are described briefly here.

SIRS Model: Because the relationship between absolute humidity and influenza incidence is less clearly understood in the tropics [42,46,47], and because humidity data quality in the tropics is poor, we use a simplified model for these countries that does not incorporate absolute humidity forcing. Here, *R*_*0*_ is defined simply as *βD*, and neither *β* nor *R*_*0*_ change over time. Thus, one less parameter (*R*_*0*_ vs. *R*_*0max*_ and *R*_*0min*_) is fit by our model-data assimilation system when simulating influenza transmission in the tropics. Initial values of *R*_*0*_ range from 0.8 to 2.2.

Data Assimilation Methods: Because influenza does not exhibit a coherent seasonal pattern in the tropics, model fitting cannot be performed as described above for temperate regions. Rather, fitting is performed continuously, beginning with the first available observation (as early as October 2010) and ending with the last, as described in [18].

Forecast Generation: Because the duration of influenza outbreaks in the tropics cannot be known in real time, forecasts are not run through the end of an outbreak period, as in temperate countries. Rather, forecasts for a given week are run 40 weeks into the future. As in temperate regions, we perform 5 simulations of 300 ensemble members each.

### Choice of scaling factors

As described above, model output represents true influenza incidence per 100,000 population. Our data, on the other hand, are obtained by multiplying nonspecific syndromic data by influenza positivity rates among those who actively seek medical care. Furthermore, the majority of countries included in the WHO data provide no information on the total number of patients seen or the size of the catchment areas from which data were obtained. Thus, our data represent counts, not rates. In order to properly use the EAKF as described above, we must therefore first scale the data such that they are compatible with the model-simulated state space. In effect, the scaling factors map the observed syndromic+ data to the model state space. Scaled data, thus, represent the estimated number of syndromic+ cases per 100,000 population, and can be used for data assimilation. Model output—the simulations and forecasts—can then be scaled back to their original units (e.g. ARI+) for use by individual country public health departments.

Our previous work has shown that SIRS simulations perform optimally when 15–50% of a model population of 100,000 is infected over the course of a modeled epidemic. Therefore, scaling values, γ, for each country were determined by first calculating the range of scaling values yielding a total attack rate between 15% and 50% for each season, *i*, ([γ_15, i_, γ_50, i_]), then choosing a single country-specific scaling value based on the following rule:

$$\gamma =\;\left\{\begin{array}{cc} if\;\exists \;\gamma \;\in \;\mathbb{R}\;:\;\gamma_{15,\;i}<\;\gamma <\gamma_{50,\;i}\;\forall \;i: & max_{i=1}^{n}\left(\gamma_{15,\;i}\right)\\ else: & min_{i=1}^{n}\left(\gamma_{50,\;i}\right) \end{array}\right\}$$ (4)

Although forecasts in the tropics were run continuously rather than by season, scaling factors for tropical countries were determined similarly using influenza outbreaks as identified under “Delineation of Influenza Seasons” above.

Scaling values were allowed to vary by country, but not by season: that is, for each country, a single scaling value was chosen and used in retrospective forecasts of all available seasons. As scaling factors are controlling for differences in rates of seeking medical attention, size of the catchment area from which influenza data are collected, and overall population size by country, they vary substantially, from 0.004 in Mexico to 374 in Peru.

### Forecast accuracy and comparison

Forecasts were evaluated based on their ability to accurately predict outbreak peak timing (the week with the highest number of influenza cases), peak intensity (the number of influenza cases at the peak), and onset timing (the first of three consecutive weeks with influenza activity over some baseline value). Onset baseline values were chosen as 500 simulated cases for temperate countries, and 300 cases for tropical countries (see S1 Text). A forecast was considered accurate for peak timing and onset timing if the predicted value was within one week of the observed, and for peak intensity if the predicted influenza case count was within 25% of the observed. These thresholds, particularly the 1 week cutoff for peak timing accuracy, have been routinely used both in our past work [13,14,19,43,45,48,49] and in evaluating forecasts submitted to the Centers for Disease Control and Prevention’s (CDC) Predict the Influenza Season Challenge [15], allowing for comparison between the results of this work and past work. If the mode predicted onset timing is NA (no outbreak), predicted peak timing, peak intensity, and onset timing were set to NA, and the forecast was removed from consideration.

Forecast accuracy was compared for temperate vs. tropical regions, as well as within temperate regions by hemisphere, region, data type, season, and scaling, and within the tropics by region, data type, and scaling. Because, in real time, the actual time to peak is unknown, we evaluated forecast accuracy by predicted lead time (i.e. the difference between the week at which a forecast is initiated and predicted peak timing). For most analyses, forecast accuracy was assessed at predicted lead weeks -6 to 4 (i.e. six weeks before the predicted peak through four weeks after). Comparisons were made for each individual variable using generalized estimating equations (see S1 Text for more details). To assess whether the effects of the explanatory factors change over time, GEE models were also run restricting the data to either before or after the predicted peak. Seasons with no identified onset (in other words, where no outbreak occurred) were removed before analyzing forecast accuracy. Additionally, because individual outbreaks within tropical countries are identified during the forecasting process, and therefore were not checked for quality previously, outbreaks where a) five or more consecutive weeks of data were missing; or b) data collection for an outbreak began at the outbreak peak were removed from tropical countries’ results before GEEs were run.

To assess the impact of including humidity forcing in the temperate models, we generated an additional set of forecasts for the temperate regions, this time without including humidity forcing in the model structure (see S1 Text). This resulted in two distinct forecasts for each country, season, start week, and run: one incorporating humidity data and one not. In order to fully take advantage of this paired design, forecast accuracy was compared by observed lead week using the exact binomial test. Because individual comparisons were made for each lead week, we applied a Bonferroni correction and considered differences to be statistically significant when p-values were less than 0.0045 (p = 0.05 / 11). Unlike in previous analyses, rather than removing forecasts predicting no onset, we considered these forecasts to be “inaccurate.” This was done to avoid ignoring pairs of forecasts where one failed to recognize an oncoming outbreak but the other accurately predicted peak timing or intensity.

Sensitivity analyses were performed to test how forecast accuracy changes as a function of EAKF observational error variance, onset baseline value, scaling, and accuracy metric. Findings from these sensitivity analyses broadly agree with the results presented here (results in S1 Text).

## Results

### Influenza data

Retrospective forecasts were performed using syndromic+ data from 64 countries, of which 18 were classified as tropical. In the temperate regions, data were available for between 2 and 7 seasons, with each country contributing an average of 6 seasons of data (data in S2 Table). In the tropics, data were available for between 29 and 345 weeks (mean = 166 weeks; median = 140 weeks). In the northern temperate region, onset timing occurred between weeks 45 and 64, and peak timing occurred between weeks 48 and 67. In the southern temperate region, these values were weeks 23 and 33 for onset timing and 29 and 38 for peak timing.

### Forecast feasibility

**Fig 2:**
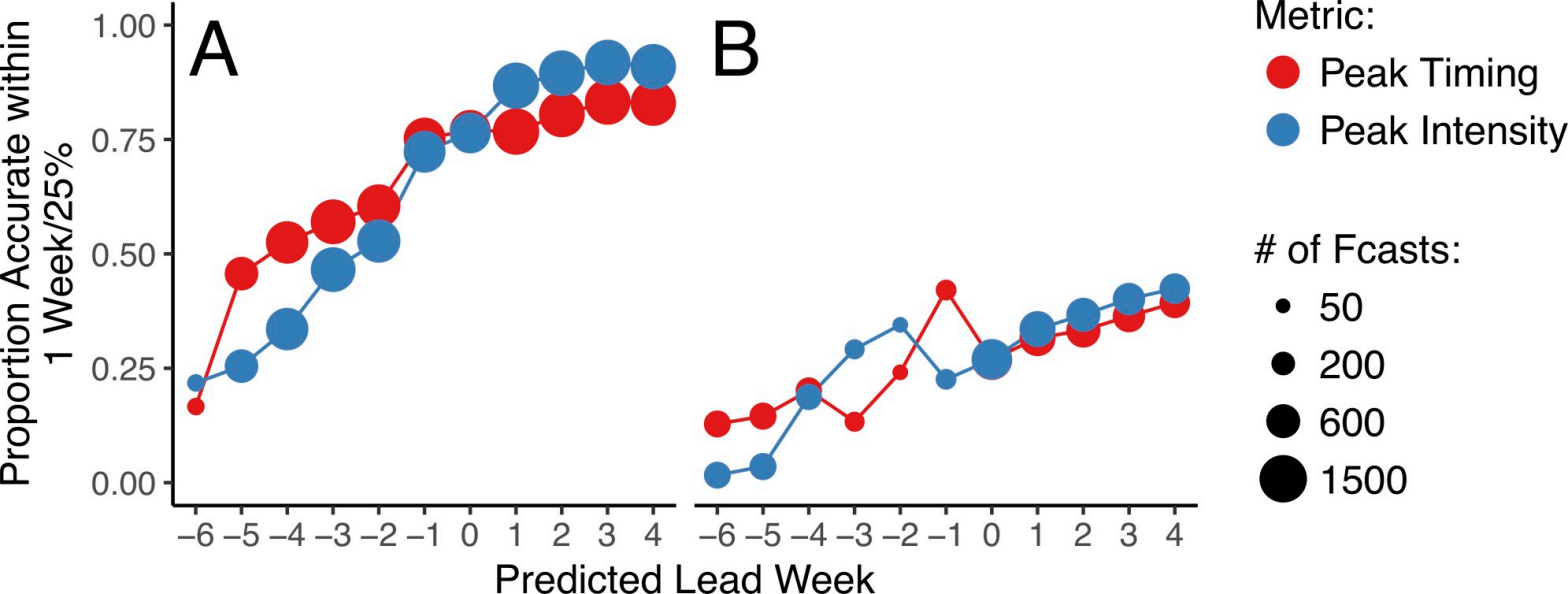
Peak timing and intensity forecast accuracy by predicted lead week. (A) Forecast accuracy in temperate regions. (B) Forecast accuracy in tropical regions. Peak timing accuracy is shown in red, and peak intensity in blue. The size of the circles represents the number of forecasts generated at a particular lead week.

Overall, we found that accurate forecasts of both peak timing and peak intensity for influenza outbreaks are possible using publicly available WHO data. In temperate regions, we were able to develop country-level, retrospective forecasts that exceeded 50% accuracy for peak timing (i.e., 50% of forecasts predicted peak timing within one week of the observed value) up to four weeks before the predicted peak, and for peak intensity (within 25% of the observed value) two weeks before the predicted peak. Forecasts exceeded 75% accuracy for peak timing one week before the predicted peak, and for peak intensity at the predicted peak week (Fig 2A). Forecast accuracy was lower in the tropics, never exceeding 50% for either peak timing or peak intensity (Fig 2B). As expected [11,12,14,17,18], forecast accuracy varied as a function of lead time, with forecasts near and after the forecasted peak typically performing better than forecasts generated several weeks before the peak. Similar patterns were seen by observed lead time, although tropical forecast accuracy was much higher after the observed peak, exceeding 70% (results in S3 Fig). Broadly, these results remained consistent after altering the cutoff point at which forecasts were considered accurate (S11 Fig), and when correlation coefficients and symmetric mean absolute percentage error (sMAPE) over the entire forecast period were assessed (S12 Fig), although forecast accuracy assessed using sMAPE was comparable between temperate and tropical regions.

For both temperate and tropical regions, forecasts of outbreak onset timing showed high accuracy post-onset, but forecasts were rarely generated in advance of the predicted onset week (Table 1). Specifically, no temperate forecasts predicted that onset would occur with more than a one week advanced lead, and very few forecasts in the tropics accurately predicted onset with more than a one-week lead. In temperate regions, onset timing accuracy (onset predicted within one week of the observed value) quickly increased and remained above 95% as soon as the predicted onset was in the past. In the tropics, accuracy reached almost 50% at the predicted onset, and remained around 65–70% for all later lead weeks.

**Table 1:** Onset timing accuracy and number of forecasts predicting any onset by predicted onset week.

Lead Week		-6	-5	-4	-3	-2	-1	0	1	2	3	4
Temperate (w/ humidity)	Accuracy	-	-	-	-	-	41.4%	87.0%	95.6%	95.7%	95.5%	95.2%
	# of Fcasts	0	0	0	0	0	29	1076	1257	1335	1319	1320
Tropical	Accuracy	13.3%	12.7%	11.1%	10.7%	6.7%	50.0%	47.2%	67.3%	70.7%	69.1%	69.1%
	# of Fcasts	165	267	305	290	104	14	339	284	300	285	285

For the tropics only, we also evaluated how often forecasts correctly recognized an existing or upcoming outbreak, without mistakenly predicting outbreaks during periods in which no outbreaks occurred. Specifically, we calculated sensitivity, specificity, positive predictive value, and negative predictive value. We found that both sensitivity (98.56%) and the negative predictive value (98.10%) were high, but that specificity (56.22%) and the positive predictive value (63.12%) were much lower. Thus, while forecasts are unlikely to predict dormancy before or during an outbreak, forecasts suggesting a current or upcoming outbreak were inaccurate more often than accurate.

#### Comparison to method of analogues

We also compared our forecasting results to results obtained using the method of analogues, a non-mechanistic forecasting method previously used by Viboud et al. to forecast influenza incidence in France [36]. In temperate countries, our mechanistic forecasting approach outperformed the method of analogues slightly for peak timing, and substantially for peak intensity before the predicted peak (S13A and S13B Fig). In the tropics, the two methods performed similarly for both peak timing and intensity (S13C and S13D Fig) before the peak, and the method of analogues performed slightly better after the predicted peak. Thus, the mechanistic forecasting methods used in this work only improve upon the analogue forecasting method in temperate regions. Additional details can be found in S1 Text, and in [36].

### Factors influencing forecast accuracy

#### Temperate vs. tropical regions

As expected, forecast accuracy was significantly lower in the tropics than in temperate regions. Overall, the odds that a forecast accurately predicted peak timing in the tropics was 0.123 (95% CI: 0.091, 0.165) times that in temperate regions, and the odds of accurately predicting peak intensity in the tropics were 0.103 (95% CI: 0.072, 0.148) times that in temperate regions. This pattern held when comparisons were restricted to predicted lead weeks of 0 and greater (i.e. forecasts predicting that the peak was either the current week or in the past; peak timing aOR = 0.115 (0.084, 0.160); peak intensity aOR = 0.070 (0.045, 0.108)).

#### Impact of humidity forcing

Inclusion of humidity forcing in the temperate region forecasts significantly increased both peak timing and peak intensity forecast accuracy prior to the observed peak, and peak timing accuracy at the observed peak (Table 2). Post-peak, no significant differences in forecast accuracy were observed by inclusion of humidity forcing.

**Table 2:** Accuracy of forecasts incorporating vs. omitting absolute humidity forcing by observed lead week for both peak timing and intensity.

Obs. Lead Week:		-6	-5	-4	-3	-2	-1	0	1	2	3	4
Timing	AH	4.8%	18.6%	36.7%	47.3%	54.4%	53.6%	55.4%	70.9%	80.2%	82.1%	82.0%
	No AH	2.0%	13.2%	32.3%	41.8%	50.4%	48.3%	50.4%	71.6%	81.4%	83.6%	83.3%
	Sig.	**	**	**	**	**	**	**				
Intensity	AH	6.9%	10.1%	18.9%	26.3%	40.3%	56.8%	70.1%	86.7%	90.2%	91.0%	90.1%
	No AH	4.2%	7.5%	14.1%	25.2%	41.6%	52.8%	68.4%	87.4%	90.4%	91.8%	91.1%
	Sig.	**	**	**			*					

* p<0.001,

**p<0.0001

#### Additional factors

Forecast accuracy was also assessed by hemisphere, region, data type, season, and scaling in the temperate regions, and by region, data type, and scaling in the tropics. Few consistent, significant relationships were found. In temperate regions, peak timing accuracy was lower for countries reporting ARI+ data vs. ILI+ data both before (aOR = 0.645, 95% CI: 0.428–0.973) and after (aOR = 0.567, 95% CI: 0.334–0.965) the predicted peak. Peak timing accuracy was highest after the peak in Eastern Europe (aOR = 2.068, 95% CI: 1.095–3.889, compared to Southwest Europe; see S1 Text for information on how countries were classified into regions). Finally, compared to countries with scaling values between 2 and 10, countries using scaling values between 0 and 0.5 performed worse for both peak timing (aOR = 0.420, 95% CI: 0.181–0.982) and peak intensity (aOR = 0.170, 95% CI: 0.051–0.568) after the predicted peak. Post-peak, countries using scaling values between 10 and 20 (aOR = 0.145, 95% CI: 0.038–0.571), 20 and 100 (aOR = 0.147, 95% CI: 0.033–0.646), and 100 and 500 (aOR = 0.229, 95% CI: 0.066–0.801) also performed significantly worse for peak intensity only. No significant differences in forecast accuracy were observed by hemisphere or season for either peak timing or intensity (results in S3 Table).

Because very few forecasts were generated prior to the predicted peak week in the tropics, it was only possible to rigorously compare forecast accuracy at and after the predicted peak. No statistically significant associations between forecast accuracy and data type, region, or scaling value were found for either peak timing or intensity in the tropics (results in S4 Table).

### Forecast calibration

It is important to consider not only how accurate forecasts are, but also forecast uncertainty. This is especially true in the case of real-time forecasting: different medical and public health responses might be affected given forecast of an 80% chance of a particular outcome rather than a 20% chance. Because each forecast is based on 300 individual ensemble members, we could assess forecast certainty through the spread of the ensemble variance, where narrower ensemble spread ideally indicated greater certainty.

**Fig 3:**
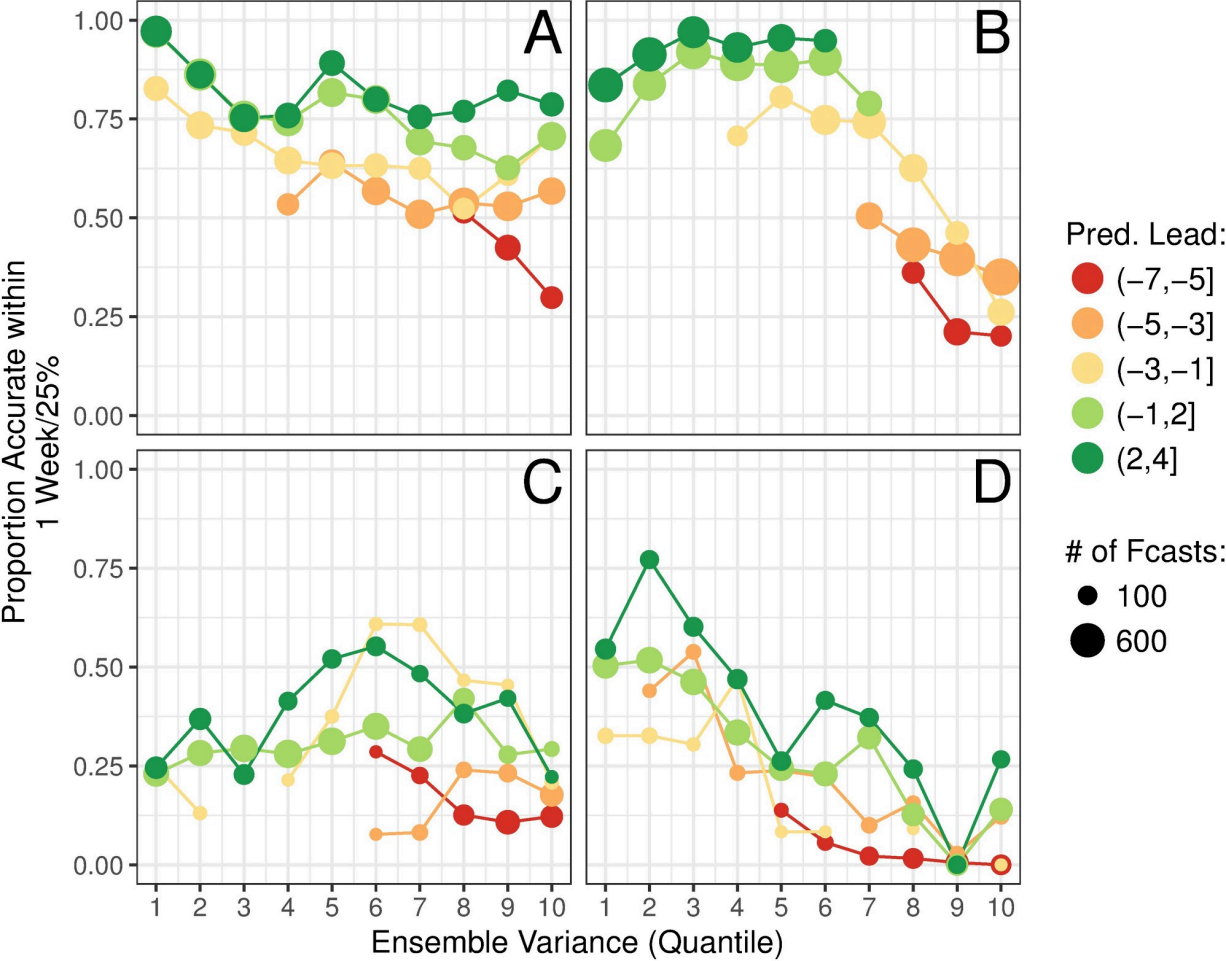
Forecast calibration as the relationship between forecast accuracy and ensemble spread. The relationship between peak timing (A and C) and peak intensity (B and D) ensemble variance and forecast accuracy by predicted lead week is shown for temperate (A and B) and tropical (C and D) regions. Point size represents how many forecasts are included in the point, and only lead week ranges with at least 100 (A and B) or 10 (C and D) forecasts were included.

Fig 3A and 3B show average peak timing and intensity forecast accuracy, respectively, for temperate regions plotted against ensemble variance (separated into 10 quantiles). For peak timing, we generally saw a slight decrease in forecast accuracy as ensemble variance increases at all predicted lead weeks, indicating that we can infer expected forecast accuracy from ensemble spread. For peak intensity, this pattern only held prior to the predicted peak. Corresponding plots for the tropics are shown in Fig 3C and 3D. For peak timing, no clear relationship existed between ensemble variance and forecast accuracy, indicating that no information about expected forecast accuracy can be inferred from ensemble spread. For forecasts of peak intensity, on the other hand, increases in ensemble variance corresponded to substantial decreases in forecast accuracy.

**Fig 4:**
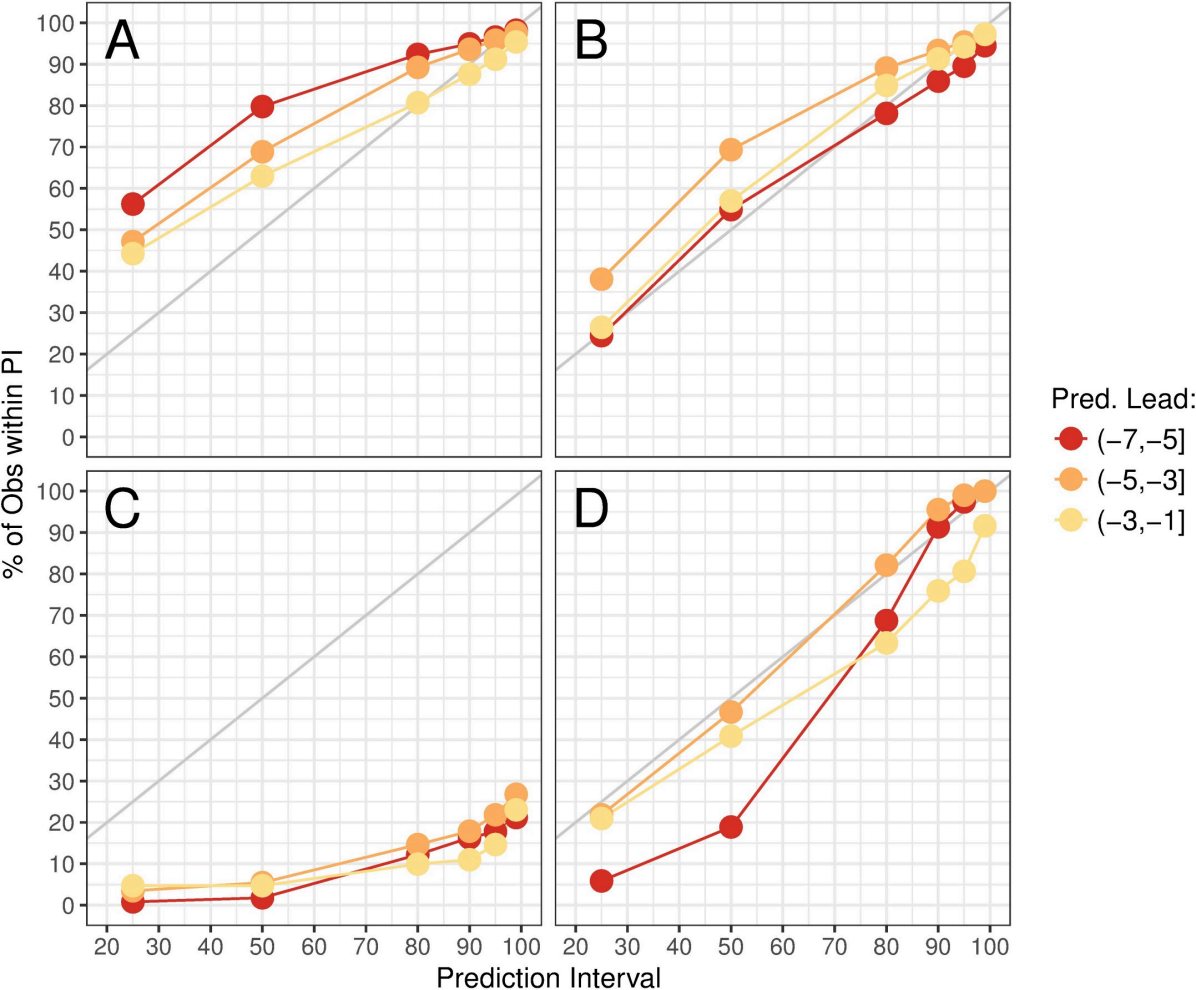
Forecast calibration as percent of observed peak timing/intensity values falling within prediction intervals. Here we show the percentage of forecasts where the observed peak timing (A and C) or intensity (B and D) value falls within the 25%, 50%, 80%, 90%, 95%, and 99% prediction intervals of 300 ensemble members by predicted lead week for temperate (A and B) and tropical (C and D) regions. The gray line represents the expected case in which exactly 25% of observations are falling within the 25% prediction interval, and so on.

We also explored how often the observed peak timing and intensity fall within certain prediction intervals of ensemble spread prior to the predicted peak (Fig 4). In a well-calibrated forecast, we expect that the observed intensity will fall within the *n*th% prediction interval *n*% of the time. Overall, forecasts appeared to be well calibrated for both peak timing and intensity in temperate regions at all lead times, although prediction intervals tended to be too wide for peak timing, especially several weeks before the peak. In the tropics, peak intensity forecasts appeared well calibrated, while peak timing forecasts rarely included the observed peak timing.

Further exploration of forecast calibration can be found in S10 Fig.

## Discussion

While skillful forecasts of influenza activity have repeatedly been shown to be possible [11–17,36,49–51], few attempts to forecast non-pandemic influenza outbreaks in areas other than the US have been made. Here, we use publicly-available syndromic and virologic data to generate retrospective forecasts of influenza transmission at the country scale for 64 countries in both temperate and tropical regions. We find that accurate and well-calibrated forecasts are possible in temperate regions. On average, peak timing and peak intensity of outbreaks can be predicted within 1 week and within 25% of the observed values, respectively, over 50% of the time starting four (peak timing) and two (peak intensity) weeks before the predicted peak, although forecast accuracy differs substantially by country (results in S2 Fig). These results are broadly consistent with past forecasting results in various US cities [11,12] as well as in Victoria, Australia [13,14], indicating that the larger spatial scale employed here does not substantially compromise forecast accuracy.

As expected, forecasts were both less accurate and less well calibrated in the tropics. Typically, peak timing and intensity could not be predicted within 1 week or 25% of observed values, respectively, until after the peak was estimated to have occurred, and the proportion of forecasts achieving these accuracy levels never exceeded 50%, even several weeks after the predicted peak. For peak timing in particular, prediction intervals based on 300 ensemble runs rarely included the observed peak week, and ensemble variability was not strongly associated with forecast accuracy, making forecast calibration challenging. Finally, while sensitivity and the negative predictive value were high, specificity and the positive predictive value were low, indicating that forecasts often predicted outbreaks when no outbreaks occurred in reality. Previously, Yang et al. produced forecasts of non-pandemic influenza in Hong Kong using methods similar to those employed here, and found that both peak timing and intensity accuracy reached 50% by lead week 0 [18]. On average, our tropical forecasts perform more poorly, although we note that, as in temperate regions, forecast performance varied substantially by country (results in S2 Fig). It is possible that the data for many of the tropical countries used in forecasting here are simply noisier than the Hong Kong data. If so, this issue may be difficult to surmount without changes in surveillance methods: smoothing our tropical data using a simple moving average over three weeks did not substantially improve forecast accuracy (results in S1 Text and S8 Fig), nor did performing model fitting and forecasting by individual outbreak instead of continuously across multiple outbreaks (results in S1 Text and S9 Fig). We also emphasize that, while our method is well tested in temperate regions, very little forecasting has been performed in the tropics. Our results do not suggest tropical countries will always yield forecasts with low accuracy, simply that the combination of data and methods applied in temperate countries may be insufficient.

Unlike peak timing and intensity, onset timing was not accurately predicted before outbreak onset in either temperate or tropical regions. This poor performance is likely due to a lack of signal in the data prior to the start of an outbreak, and is not surprising. Past work has shown that models including travel between US states [19] and boroughs of New York City [52] significantly improve forecast accuracy, particularly onset timing accuracy. Future work will incorporate travel between countries in the model, allowing forecasts of onset timing in a given country to be informed by signal from connected countries in which an outbreak has already begun. While a variety of models exist for forecasting the spatial dynamics of influenza transmission [53–55], we believe that our approach, in which a model is iteratively fit to influenza observations, can offer significant improvements.

Significant differences in forecast accuracy were observed by a variety of factors for both peak timing and intensity. In temperate regions, forecasts of peak timing are less accurate for countries reporting ARI data than for those reporting ILI data. Because ARI is a less specific measure than ILI, these data tend to be noisier. This, in turn, likely contributes to the lower forecast accuracy. We also observe lower peak timing accuracy with particularly small scaling values, and lower peak intensity accuracy with particularly small or large scaling values, at least after the peak.

Including absolute humidity forcing in our models improved temperate forecast accuracy prior to the observed peak, with no differences observed post-peak. These results are consistent with the results of a recent paper by Shaman et al., which found that, on average, including absolute humidity forcing improved forecast accuracy in 95 US cities before the predicted peak [43]. Given the large spatial and latitudinal scale of several of the countries examined here, it is interesting that mean country-level absolute humidity still significantly improves forecast accuracy. Our results suggest that absolute humidity forcing should continue to be included in models forecasting influenza in temperate regions, even when humidity must be averaged over large regions. Furthermore, given evidence that climatic factors such as absolute and relative humidity [56–59] and precipitation [47,56,57,59] may influence influenza transmission in the tropics and subtropics, future work should consider how climatic factors may be incorporated into model fitting and forecasting outside of the temperate zones.

Despite the novelty of this work, we are cognizant of several important limitations. First, our data exhibit a strong spatial bias, with little to no representation in Africa and South America. Information on influenza dynamics in general are particularly lacking in the tropics, which precludes forecasting. As always, forecast accuracy findings may also be dependent on the choice of accuracy metrics, although we note that our results are robust to various accuracy cutoffs, as well as to choice of alternative accuracy metrics (see S1 Text and S11 and S12 Figs). Furthermore, our method outperforms the method of analogues, a robust non-mechanistic forecasting method [36] in temperate regions (S1 Text and S13 Fig).

Additionally, as mentioned in the Methods, data do not perfectly reflect reality. All syndromic data types include some people with non-influenza respiratory conditions, and exclude those with influenza who do not seek treatment or meet specific criteria. Multiplying by percent of tests positive for influenza only partially mitigates these issues. In particular, differences in noisiness between different data types persist. Information on the size of the catchment area from which data were obtained is also largely lacking, so forecasts must be generated based on raw counts rather than rates. This leads to substantial variability in case counts by country. While this can be partially compensated through the use of scaling factors, it is crucial that these values are chosen appropriately [13]. As we base scaling values on past data, forecast accuracy may therefore be compromised when data are not available for several past seasons. Furthermore, if new countries begin submitting influenza data, real-time forecasts cannot be generated immediately, as at least one full season or outbreak must pass before an appropriate scaling can be calculated.

Finally, all forecasts at this point have been generated at the country level. Thus, while our results and future real-time forecasts may be of public health relevance for smaller countries, they are likely to provide less actionable results for much larger countries, such as Russia or Brazil. Future work should attempt to incorporate subnational data, where available. In addition to increased public health relevance, we may also expect forecast accuracy to improve when smaller subunits within a country are used for forecasting. We note, however, that real-time forecasts are only plausible when data are submitted in a timely fashion. At present, this occurs for most of the northern hemisphere temperate countries included in this study, but is uncommon in southern hemisphere temperate countries and for countries in the tropics and subtropics.

### Conclusions

We have shown that, in temperate regions, accurate and well-calibrated retrospective forecasts of seasonal influenza activity are feasible. Work is currently being conducted to determine whether real-time forecasts are similarly feasible, and future work will incorporate travel between countries with the goal of improving forecast accuracy, particularly onset timing accuracy. Although this work is at an early stage, we note the importance of eventually incorporating forecasts into medical and public health decision-making. Accurate real-time probabilistic forecasts have the potential to inform decisions such as antiviral stockpiling by governments or staff and bed management by hospitals, preventing morbidity and mortality. Therefore, it is critical that these forecasts not be produced solely as an academic exercise.

## Supporting information

S1 Text Supplementary methods and results. (PDF)

S1 Dataset Processed syndromic+ data from 64 countries. The data provided here are not yet scaled by the chosen scaling factors.
(CSV)

S2 Dataset Processed absolute humidity climatologies for 46 temperate countries. Each column represents the average specific humidity for days 1–365 of the year for a single country.
(CSV)

S1 Fig Syndromic+ data by region. All data are divided by the maximum observed incidence in a given country since the 2010–11 season.
(PDF)

S2 Fig Forecast accuracy by predicted lead week by country. (A) Peak timing accuracy. (B) Peak intensity accuracy. NA values are represented by gray boxes.
(TIF)

S3 Fig Peak timing and intensity forecast accuracy by observed lead week. (A) Forecast accuracy in temperate regions. (B) Forecast accuracy in tropical regions. Peak timing accuracy is shown in red, and peak intensity in blue.
(TIF)

S4 Fig Forecast accuracy by OEV denominator choice. Peak timing (A and C) and intensity (B and D) accuracy for different OEV denominators in temperate (A and B) and tropical (C and D) regions.
(TIF)

S5 Fig Forecast calibration by OEV denominator choice. Peak timing (A and C) and intensity (B and D) calibration by OEV denominator in temperate (A and B) and tropical (C and D) regions.
(TIF)

S6 Fig Forecast accuracy by choice of onset value. Onset timing accuracy by choice of onset value in temperate (A-C) and tropical (D-F) regions.
(TIF)

S7 Fig Forecast accuracy by choice of scaling rule. Peak timing (A and C) and intensity (B and D) accuracy by choice of scaling rule in temperate (A and B) and tropical (C and D) regions.
(TIF)

S8 Fig Forecast accuracy for the tropics using smoothed and unsmoothed data. (A) Peak timing accuracy. (B) Peak intensity accuracy.
(TIF)

S9 Fig Forecast accuracy for individual tropical outbreaks. (TIF)

S10 Fig Histograms of peak timing and intensity forecast error. Distribution of peak timing (A and C) and intensity (B and D) errors relative to observed for temperate (A and B) and tropical (C and D) regions. To make peak intensity errors comparable between countries, errors are standardized by the observed peak intensity for a given country and season.
(TIF)

S11 Fig Forecast accuracy using alternative accuracy cutoffs. Percent of forecasts accurately predicting peak timing and intensity in temperate (A and C) and tropical (B and D) countries. (A and B) Forecasts are considered accurate if they predict peak timing exactly, and predict peak intensity within 12.5% of the observed value. (C and D) Forecasts are considered accurate when forecasts are within 2 weeks of the observed peak timing and 50% of the observed peak intensity.
(TIF)

S12 Fig Forecast accuracy using correlation coefficients and symmetric mean absolute percentage error (sMAPE). Ranges of correlation coefficients (A and B) and sMAPE (C and D) for temperate (A and C) and tropical (B and D) countries. Points represent median values, and error bars show the 95% credible interval. Point size represents the number of forecasts contributing data to the point in question.
(TIF)

S13 Fig Forecast accuracy using the method of analogues. A comparison of peak timing (A and C) and peak intensity (B and D) accuracy in both temperate (A and B) and tropical (C and D) countries between the methods described in the main text (red) and the method of analogues (blue).
(TIFF)

S14 Fig Inferred model states and parameters. Ranges for *S0*, *R*_*e*_, *R*_*0*_, *D*, and *L* by temperate versus tropics designation (A), hemisphere (B), and data type separated by temperate (C) and tropical (D) regions.
(TIF)

S15 Fig Ranges of *R*_*0max*_ and *R*_*0min*_ by latitude. Distribution of inferred values for *R*_*0max*_ (A and B) and *R*_*0min*_ (C and D) by latitude (absolute value), defined as the latitude at the country’s centroid (A and C) or the latitude of the country’s capital (B and D). Values derived from temperate countries are shown in blue, and values from countries in the tropics are in red.
(TIF)

S16 Fig Posterior visualizations. Mean posterior fit for 5 models runs of 300 ensemble members each for Norway, Poland, Italy, Mexico, and Ecuador. Fit is plotted for the 2015–16 season for the temperate countries, and for the entire duration of the available data for Ecuador. Observed data are plotted as black x’s, while the posterior model fit is plotted in blue.
(TIF)

S17 Fig Temperate forecast visualizations. Forecast trajectories for Norway, Poland, Italy, and Mexico for the 2015–16 season. Forecasts are presented starting 6 weeks prior to the observed week through 2 weeks after the peak. Black x’s represent observed data, blue lines show model incidence during the training period, and red lines represent forecast trajectory. For each forecast, the 5 runs are shown separately.
(TIF)

S18 Fig Tropical forecast visualization. Forecast trajectory for the fifth recorded outbreak in Ecuador in our dataset. Forecasts are presented as in S17 Fig. Because tropical forecasts were generated by fitting the model to the observations continuously, rather than by season, model fitting for all data prior to the fifth outbreak in Ecuador is shown.
(TIF)

S19 Fig Forecast accuracy for the 2009 influenza pandemic. Peak timing (red) and intensity (blue) accuracy for forecasts of the 2009 pandemic in temperate (A) and tropical (B) countries.
(TIF)

S1 Table Countries used for retrospective forecasting, by region, data type, and scaling. (PDF)

S2 Table Countries and seasons used for retrospective forecasting. (PDF)

S3 Table Peak timing and intensity accuracy overall, before the predicted peak, and at or after the predicted peak in temperate regions by hemisphere, season, region, data type, and scaling. Cells shaded in green indicate improved forecast accuracy over the reference level, while cells shaded in red indicate reduced accuracy.
(PDF)

S4 Table Peak timing and intensity accuracy at or after the predicted peak in the tropics by region, data type, and scaling. Cells shaded in green indicate improved forecast accuracy over the reference level, while cells shaded in red indicate reduced accuracy.
(PDF)

S5 Table Inferred model states and parameters for all countries and outbreaks. Results for each of 5 individual runs for each country and outbreak are included. For tropical countries, the “season” column contains a number from 1 to *x*, where *x* is the total number of outbreaks (as defined in the main text) observed in a country over the considered years, denoting which of the *x* sequential outbreaks the inferred values refer to.
(CSV)
