# Supplementary material for: Development and validation of influenza forecasting for 64 temperate and tropical countries
Source: PLoS Comput Biol. 2019 Feb 27;15(2):e1006742. doi: 10.1371/journal.pcbi.1006742 (PMC6411231; doi:10.1371/journal.pcbi.1006742)
Supplement: S1 Text — (PDF) [file pcbi.1006742.s001.pdf]

S1 Text: Supplementary methods and results.

SUPPLEMENTARY METHODS

Influenza Data Processing

Both syndromic and virologic data were downloaded from the WHO in April 2018.

When a country reported several different types of syndromic data, we used the data type that was most consistently reported across all seasons. When two data types were reported with roughly equal frequency, we favored ILI, as it is more specific than ARI, but includes a wider population than SARI or pneumonia. Data were also examined visually for signal; if one data type appeared to produce a much smoother signal than another, it was chosen for use in forecasting. France reported ARI data prior to the 2014-15 influenza season, but switched to ILI data for the 2014-15 season and all subsequent seasons; all other countries have favored the same data type or types over time.

Overall, 38 countries reported ILI, 17 reported ARI, 6 reported SARI, one (Honduras) reported pneumonia, one (Canada) reported ILI rates rather than counts, and one (France) changed preferential data types during the period spanned by the data (from ARI to ILI) (S1 Table). SARI and pneumonia were only preferentially reported from tropical countries. Definitions for all 4 syndromes, however, are not standardized, and therefore the specific definitions used vary by member state. Broadly, ILI refers to a respiratory illness involving fever and cough, whereas ARI is less strict and captures patients with at least one of several respiratory symptoms. A diagnosis of SARI, meanwhile, requires hospitalization [1].

Virologic data consisted of the number of tests positive for any influenza strain, as well as the number of tests processed and reported. The proportion of tests positive for influenza was calculated by dividing the number of positive tests by the number of tests processed; when no information regarding tests processed was available, the number of positive tests was instead divided by the number of tests reported. If the resulting proportion exceeded one, the data point was removed.

Countries were maintained in the data set if they had good quality syndromic and virologic data for at least one season. In the temperate regions, good quality data were

defined as data for which fewer than one third of all available seasons met the removal criteria described in the main text (i.e. at least 5 consecutive missing data points near the peak); in other words, temperate countries were maintained in the data set if over two thirds of available seasons could be used for forecasting. In the tropics, countries were removed from consideration if: 1) over 33% of total observations or over 5% of observations during outbreaks were 0, or 2) if the highest peak was over 15 times higher than the lowest peak (i.e., if the data showed an unrealistic amount of variation from outbreak to outbreak).

Finally, several countries were located between temperate and tropical regions in the subtropics, whereas others spanned both temperate and tropical regions. To classify these countries as “temperate” or “tropical” for the sake of this study, we therefore considered whether past influenza outbreaks exhibited a marked seasonal signal consistent with temperate influenza activity. If outbreaks occurred once a year and strictly within the seasons defined for temperate regions (weeks 40 to 20 in the northern hemisphere, or weeks 14 to 46 in the southern), the country was classified as “temperate;” otherwise, we classified it as “tropical.”

All data used for forecasting are described in S1 and S2 Tables, and visualized in S1 Fig. The fully processed data are provided as S1 Dataset. Note that these data are not yet multiplied by the relevant scaling values.

Humidity Data Processing

We processed the raw data for use in our models as follows. First, daily averages for each 1°x1° grid cell were calculated to yield daily time series of specific humidity. Next, we searched the humidity data visually for anomalies. Three major anomalies were found: 1) in some grid cells, humidity increased substantially for the years 1994-1998; 2) in some grid cells, humidity was anomalously low in either 1999 or 2003-2004; and 3) in several countries, primarily Australia and countries in Eastern Europe, a sharp increase in humidity was observed throughout the majority of 1997, excepting the summer. The first two anomalies were addressed by removing any years for which yearly average specific humidity was over 1.5 times the 75th percentile or less than 0.65 times the 25th percentile of yearly average specific humidity for a given grid cell. The

third anomaly was identified by visual inspection, and the year 1997 was removed from affected grid cells. A total of 469 grid cells were affected by the first anomaly, 44 by the second, and 1270 by the third; additionally, two grid cells from Chile were removed entirely because data shifted substantially upward and downward over time. Overall, 1800 grid cells had at least some data removed due to anomalies, leaving 6240 grid cells with no anomalies during the 20-year record.

Twenty-year climatologies for each grid cell were then generated by averaging daily specific humidity on each of 365 days across twenty years. Note that, due to removal of anomalous data by year, many grid cells yielded 12 to 19 year climatologies. Each grid cells was then assigned to one or more countries using the Clip tool in QGIS 2.18.2. Grid cells belonging to more than one country were delegated proportionally to all countries with which they overlapped. Finally, climatologies were aggregated to the country level by taking an average of the climatologies for all grid cells assigned to a given country, weighted by the proportion of the grid cell situated within the country in question. The processed humidity data are provided as S2 Dataset, where each column represents the average specific humidity for days 1 – 365 of the year for a single country.

Ensemble Adjustment Kalman Filter

Observational Error Variance: As described in the main text and above, both syndromic and virologic data are subject to error from a variety of sources, and thus deviate from the number of true influenza cases. However, the extent of this error is unknown. In order to properly use the EAKF, we must therefore specify some degree of error in our observations. We account for this by calculating observational error variance (OEV), defined as:

$$OEV_t = 1\times10^5 + \frac{\left(\sum_{j=t-3}^{t-1} \frac{O_t}{3}\right)^2}{c}$$

where O_t is the observed syndromic+ data at time t and c can be altered to modify the magnitude of the prescribed error, with lower values of c corresponding to higher overall

error in the observations. All forecasts described in the main text were run with *c* equal to 1. Results of sensitivity analyses using *c* = 10 are presented below.

Filter Divergence: One prominent issue with the EAKF is that of filter divergence, in which, following assimilation of multiple successive observations, the variance of the model ensemble decreases, and thus confidence in the model estimates increases to the point where the observations are essentially ignored. To prevent filter divergence in temperate regions, we multiplicatively inflate the prior model variance by 1.03 times before assimilating each new observation [2,3]. In the tropics, where model fitting is performed over several years, filter divergence is likely to be a more substantial issue than over the shorter, seasonal time periods modeled for temperate countries. As in the temperate regions, we address filter divergence by multiplicatively inflating the ensemble variance by 1.03 at each time step. Additionally, per Yang et al. [3], if the model diverges from an observation by more than 20% at a given time step, we reinitialize the model completely by choosing new initial states and parameters at that time step. These methods are described in more detail in [3].

Retrospective Forecast Generation (Temperate without Humidity Forcing)

To generate retrospective forecasts in temperate regions with no absolute humidity forcing, we used the same SIRS model as for tropical forecasts. As with all other forecasts, we ran 5 simulations of 300 ensemble members each.

Comparing Forecast Accuracy

To compare forecast accuracy in the temperate and tropical regions, as well as by hemisphere, region, season, data type, and chosen scaling value, we used generalized estimating equations (GEEs) controlling for predicted lead week as a categorical variable with week 0 as the reference level. GEEs were chosen for their ability to control for temporal autocorrelation within each country and season pair, as the accuracy of successive weekly forecasts in a given country are temporally autocorrelated. Further, GEEs were chosen over mixed effects models in order to estimate overall effects rather than impact on individual forecasts. An autoregressive

AR(1) working correlation matrix was assumed. The five replicate forecasts produced for each country, season, and start week represent an additional layer of clustering in our results. To control for this, we randomly permuted the results 100 times, each time choosing a single run (among the 5 replicates) for each country, season, and start week (or, in the tropics, each country, start week, and individual outbreak). Final results were drawn from the median coefficients and standard errors of these 100 permutations. Results for all tested factors for both temperate and tropical regions can be found in Tables S3 and S4.

Tropical Data Smoothing

We hypothesized that forecast accuracy in the tropics could be improved by smoothing the syndromic+ data, which was typically substantially noisier than the temperate data (S1 Fig). In order to test this hypothesis, we applied a simple moving average to the tropical data. Because in real-time forecasting no data beyond the current forecast week are available, we averaged the data for a given time point with the data from the previous two weeks to create a 3-week moving average. We then ran retrospective forecasts as described in the main text using the smoothed data.

Retrospective Forecast Generation by Tropical Outbreak

To assess the role of sporadic outbreak timing on forecast accuracy in the tropics, we also ran tropical forecasts for each outbreak individually, similar to how forecasting was performed in temperate regions. Outbreaks for each tropical country were identified as described in the main text. We then added eight weeks to the beginning and end of each identified outbreak period, before performing forecasts as described in the main text for temperate regions, considering each outbreak as a "season." Specifically, fitting began eight weeks before the identified outbreak onset, and forecasts were generated starting 3 weeks before outbreak onset (corresponding to 5 weeks of training data, as in the temperate regions) through 4 weeks after the outbreak had ended. We note that, in temperate countries, model fitting started an average of 13 to 14 weeks prior to outbreak onset. However, because outbreaks in the tropics often happen in rapid succession, it was not possible to include this amount of

lead time around the outbreak periods without also including substantial portions of other "outbreaks."

Sensitivity Analyses for Timing/Intensity Accuracy Cutoffs

Because our conclusions regarding forecast accuracy are dependent on the ranges of predicted timing and intensity values that we consider to be "accurate," we also assessed forecast accuracy using alternative accuracy definitions, one stricter and one more lenient. Specifically, we considered forecasts of peak timing to be accurate (a) only when the forecasted peak timing equaled the observed peak timing exactly, or (b) when the forecasted peak timing was within 2 weeks of the observed peak timing. For peak intensity, we considered forecasts accurate (a) when the forecasted value was within 12.5% of the observed value, or (b) when the forecasted value was within 50% of the observed value.

Alternative Forecast Accuracy Metrics

In addition to the peak timing, peak intensity, and onset timing accuracy metrics defined in the main text, we also assessed forecast accuracy over the duration of the forecast using correlation coefficients and the symmetric mean absolute percentage error (sMAPE). These metrics are calculated by comparing forecast influenza incidence from the time of forecast start until 10 weeks post peak with the observed influenza syndromic+ data over the same time period. This time period was chosen because, beyond 8 weeks post observed peak, syndromic+ case counts tend to be low, zero, or missing, precluding meaningful error measurements. We additionally removed any forecasts with fewer than four non-NA data points. sMAPE is defined as:

$$sMAPE = \frac{100\%}{T}\sum_{t=1}^{T} \frac{|F_t - O_t|}{(|O_t| + |F_t|)/\,2}$$

where T is the number of weeks forecasted, O_t is the observed syndromic+ value at time t, and F_t is the forecasted influenza incidence at time t [4]. We chose to use sMAPE rather than the more commonly used root mean square error (RMSE) because, unlike RMSE, sMAPE controls for the difference in the magnitude of the observed data both at

different points in an outbreak, as well as between different countries in the dataset. Also, unlike MAPE, sMAPE is not highly biased toward forecasts that undershoot observed values [4].

To test whether significant differences in forecast accuracy exist between temperate and tropical regions, we performed Kruskal-Wallis rank sum tests at predicted lead weeks -6 through 4. Because 5 individual runs were performed for each country and season, we randomly chose a single run for each country and outbreak combination 20 separate times, similar to the process described in the main text for comparing forecast accuracy. If p-values were below 0.0045 (0.05 / 11; p = 0.05 with Bonferroni correction for the 11 distinct lead weeks) for at least 50% of randomly selected run combinations, we considered there to be a significant difference in value for that lead week.

Method of Analogues

We further compared our mechanistic forecasting results with results obtained using the method of analogues [5]. Explicit methodological detail can be found in [5]. Briefly, the method involves searching through the entire time series of each country for a given number of vectors, or “nearest neighbors,” that most closely match the data at the time at which a forecast is desired. We performed the method for each country individually at each time point using two nearest neighbors of length four. These nearest neighbors were drawn from previous seasons in the temperate regions, and from any previous data in the tropics; in other words, neighbors from the current season itself were not permitted when forecasting in the temperate regions. Additionally, because missing data were common in our dataset, we limited forecasting with the method of analogues to forecast start weeks where at least two of the preceding three weeks had data. Finally, as this method relies on patterns observed in past data, we do not begin forecasting until two full seasons (temperate) or outbreaks (tropics) have occurred. We note that this precludes forecasting in several tropical countries (Bangladesh, Bolivia, Brazil, Honduras, Indonesia, Kenya, and Madagascar). In order to fairly compare the method of analogues with our methods as described in the main text, we remove forecasts from our main results accordingly for this analysis only.

Data Quality Metrics

In general, we expect that forecast accuracy will be higher when data of better quality are used for model fitting. To test whether this was the case in this study, we calculated three measures of data quality:

1) The proportion of weeks within seasons (temperate countries; weeks 40 to 19 in the northern hemisphere and weeks 14 to 45 in the southern hemisphere) or outbreaks (tropical countries; outbreaks extended from the end of a previous outbreak to the next outbreak endpoint, so as to include both the outbreak itself and the most proximal training data) for which no data were available were calculated overall for each country, as well as by season for temperate countries.
2) Data signal smoothness was calculated using lag-one autocorrelation.
3) The extent to which a country sampled for influenza was estimated by comparing the number of virologic samples taken each week within the influenza season (temperate) or throughout the year (tropics) to the country's total population size.

These measures were then compared to overall average peak timing and intensity accuracy by country (and by season, for temperate countries and measure 1) using Kendall's rank correlation.

Inferred Model States and Parameters

As described in the main text, model state variables (the number susceptible and infected) and parameters (R_{0max}, R_{0min}, R_0, D, L) are inferred throughout the model fitting process. To determine whether inferred values of *S0* (the initial number of susceptible individuals in a country) and of model parameters substantially differed between temperate and tropic countries, by hemisphere, or by data type, we first limited our analysis to the training period of the final forecasts run for each country and season, as these were the model fits incorporating the greatest number of data points. Then, we calculated R_0 for temperate countries according to equation 2 in the main text. Finally, R_e, or "R effective," defined as the number of cases caused by each infected individual after taking into account the susceptibility of the population, was calculated by multiplying R_0 by S / N at each time point. The value of *S0* for a country and season (or country and outbreak in the tropics) was considered to be the maximum inferred value

of S over the course of the outbreak. R_0, R_e, D, and L were considered at the time of maximum R_e for each country and outbreak, as described in [6]. Finally, values of $S0$, R_0, R_e, D, and L were compared by region (temperate vs. tropics), hemisphere, and data type using the Kruskal-Wallis rank sum test, as described above under "Alternative Forecast Accuracy Methods." Here, results were considered significant if f p-values were below 0.05 for at least 50% of randomly selected run combinations.

Inferred Maximum and Minimum R_0 by Latitude

In each country, R_0 is allowed to vary between some maximum R_{0max} and some minimum R_{0min}, dependent on absolute humidity (see Eq. 2 in main text). R_{0max} and R_{0min} are fit separately for each country, and thus are permitted to vary. If the influence of humidity on influenza transmission acts similarly at all latitudes, we expect inferred values of R_{0max} and R_{0min} to also be similar at all latitudes.

To test this, we identified the inferred values of R_{0max} and R_{0min} for each country and season in both the northern and southern temperate regions at maximum R_e, as described in the previous section. We then compared values of these two parameters between hemispheres, as described above, as well as by latitude, using Kendall rank correlation. Again, results were considered significant if p-values were below 0.05 for at least 10 of 20 randomly selected combinations of model runs. For each country, we tested two different values for latitude: the latitude at the center of the country, and the latitude of the country's capital city. Absolute values were used so that countries in both the northern and southern hemispheres could be assessed together.

Pandemic Forecasts

We also generated retrospective forecasts for the 2009 influenza pandemic for the 34 countries (including 2 in the tropics) reporting data during this period. Because the time of pandemic emergence was not known in advance in real time, we did not begin forecasting until scaled observations exceeded 50% of the onset baseline value (250 in temperate regions and 150 in the tropics). At that point, an initial forecast was produced using 10 weeks of training data, and forecasting proceeded as described in the main text. For this reason, forecasts of pandemic onset were not possible. Note that

if the time at which syndromic+ data exceeded baseline onset was before the fifth week of data, forecasting was begun after 2 weeks of training, to avoid generating forecasts with insufficiently trained models. This was done so that model states and parameters had some degree of training before forecasts were produced. Forecasts were then generated every week until scaled observations fell below 50% of the onset baseline for >=2 consecutive weeks (which we considered the pandemic “endpoint” for a country), or until less than 4 weeks remained before the beginning of the 2010-11 influenza season (in temperate countries). Thus, the exact period over which forecasts were generated varied by country. Otherwise, forecasts were run as described in the main text.

Countries for which both syndromic and virologic data were available for the 2009 pandemic included 32 northern hemisphere temperate countries, and 2 tropical countries (Honduras and Singapore). A complete list of these countries can be found in S2 Table.

SUPPLEMENTARY RESULTS

Forecast Accuracy by Country

As observed in a previous forecasting study focusing on US cities [2] and as mentioned in the main text, forecast accuracy varied greatly by individual country (S2 Fig).

Forecast Accuracy by Observed Lead Week

When assessed by observed lead week, retrospective forecasts for temperate regions reached 50% accurate from 5 weeks prior to the peak (for peak timing) and 1 week prior to the peak (for peak intensity). Peak timing forecasts exceed 75% at 2 weeks post peak, and peak intensity forecasts exceed 75% the week after the peak (S3A Fig). These results are similar to those presented for predicted lead week in the main text.

Forecasts in the tropics exceed 50% accuracy at the observed peak for both peak timing and intensity (S3B Fig). Forecasts surpass 75% accuracy at one week post peak for peak timing, but never reach 75% accuracy for peak intensity. Thus, results are

similar to those in the main text before the peak, but demonstrate much higher accuracy post-peak.

Choice of OEV Denominator

S4 Fig compares peak timing and intensity forecast accuracy for temperate and tropical regions when *c* is set to 10, rather than 1 as in the main text, corresponding to a tenfold reduction of error in the syndromic+ observations. S5 Fig compares forecast calibration under the same circumstances. In temperate regions, setting *c* to 10 appears to have little impact on forecast accuracy. In the tropics, however, peak intensity accuracy appears substantially higher when *c* is set to 10 rather than 1. However, for both temperate and tropical regions, and for peak intensity in particular, setting *c* to 1, as presented in the main text, appears to result in better forecast calibration, i.e. the prediction intervals for peak intensity are more aligned with the spread of observations. Given our goal of producing forecasts that are both accurate and well calibrated, using a *c* of 1 appears preferable to a *c* of 10.

Choice of Onset

In the main text, we set the scaled baseline value to 500 for temperate regions and 300 for the tropics. In S6 Fig, we present the overall accuracy of onset timing forecasts when onset is set to 300, 400, or 600 in the temperate regions, and 200, 400, or 500 in the tropics. Seasons where no onset occurred were removed, and forecasts predicting no onset were counted as inaccurate. Forecast accuracy is therefore presented by lead relative to observed onset week, as predicted lead onset week does not exist when no onset is predicted. In temperate regions, there are no substantial differences in onset timing forecast accuracy by choice of onset value. In the tropics, differences are somewhat more pronounced, but the overall structure of accuracy as a function of lead is similar; note that for some baseline values more spurious predictions of no onset were generated (the smaller dots sizes in S6 Fig).

Choice of Scaling Value

In our main analyses, we systematically selected the lowest scaling values that yielded overall attack rates between 15% and 50% of the model population for all seasons, where possible. Here, we test the sensitivity of our results to this decision by essentially flipping our scaling selection rule (Eq. 4) and choosing the highest scaling values that yield the desired attack rates:

$$\gamma = \begin{Bmatrix} if\ \exists\,\gamma \in \mathbb{R} : \gamma_{15,i} < \gamma < \gamma_{50,i}\ \forall\, i: & min_{i=0}^{n}(\gamma_{50,i}) \\ else: & max_{i=0}^{n}(\gamma_{15,i}) \end{Bmatrix}$$

Results of these analyses are shown in S7 Fig. Overall, changing the selection rule has little impact on forecast accuracy.

Tropical Forecast Accuracy Using Smoothed Data

When forecasting in the tropics is performed using data smoothed with a 3-week moving average, forecast accuracy appears to improve slightly for peak intensity, but not for peak timing (S8 Fig). However, forecast accuracy remains much lower than in the temperate regions.

Retrospective Forecast Accuracy by Tropical Outbreak

When forecasts of tropical outbreaks are performed separately for each outbreak, essentially treating each outbreak as a "season," forecast accuracy before the predicted peak appears to increase slightly (S9 Fig). However, forecast accuracy remains low overall compared to temperate regions, suggesting that low forecast accuracy in the tropics is not primarily due to the irregularity of outbreaks, which prevent recurrent, seasonal model fitting and forecasting. Instead, the differences appear to be related to factors such as the high amount of noise in tropical observations.

Additional Forecast Calibration Results

In a properly calibrated forecast, we expect that errors in forecasted peak timing and intensity will display some distribution with a mean of 0. In contrast, a non-zero

mean indicates that forecasts are biased. We assess whether the forecasts generated in the main text are biased according to this measure by plotting histograms of the difference between the observed and predicted peak timing and peak intensity over time (S10 Fig). In order to standardize errors over a wide range of observed peak intensity values by country, we plot the error in peak intensity forecasts divided by the observed peak intensity, rather than simply plotting the absolute error. Using this metric, we see that good calibration is achieved in the temperate regions, particularly directly prior to the peak. Calibration appears substantially worse in the tropics, where both peak timing and peak intensity are consistently underestimated. Thus, although forecasts of peak intensity in the tropics yield informative and well-constrained credible intervals (Figs 3 and 4 in the main text), they display substantial bias.

Forecast Accuracy Using Alternative Accuracy Cutoffs

As expected, calculated forecast accuracy decreased when stricter accuracy cutoffs were employed (S11 Fig A and B) and increased when less strict cutoffs were used (S11 Fig C and D), However, observed patterns in accuracy remained the same: forecast accuracy generally increased as predicted lead week increased, and accuracy in temperate regions was noticeably higher than in the tropics.

Forecast Accuracy Using Correlation Coefficients and sMAPE

Correlations between observed and forecasted incidence were significantly higher for temperate than tropical countries for all lead weeks except predicted lead week -6, where very few forecasts were available (S12 Fig A and B). Also notable were the wide confidence intervals around correlation coefficient estimates in the tropics, with 95% credible intervals ranging from -0.68 to 0.97 (as opposed to 0.46 to 0.995 in temperate countries). sMAPE values, on the other hand, were similar in temperate and tropical regions, with no statistically significant differences observed at any predicted lead week. While we believe that the targets used in the main text (peak timing and peak intensity) represent metrics of practical importance for responding to influenza outbreaks, it is nonetheless important to acknowledge that the impact of temperate vs. tropical region on forecast accuracy is dependent on how forecast accuracy is

measured, and that tropical forecasts may perform better for other metrics not measured here.

Method of Analogues Forecast Accuracy

Results are primarily discussed in the main text. However, we note here that, because the method of analogues requires information on past outbreaks, early outbreaks could not be forecasted for any country, and several tropical countries had to be removed from consideration entirely. Before comparing to mechanistic forecasts (S13 Fig), we therefore removed any country or season that could not be forecasted using the method of analogues.

Forecast Accuracy by Data Quality

All three measures of data quality were found to differ significantly between temperate and tropical regions (Kruskal-Wallis one-way analysis of variance, $p < 0.01$ for all measures). Therefore, the relationship between these measures and forecast accuracy was assessed separately for temperate and tropical countries.

Greater smoothness of data signal was significantly associated with higher peak intensity accuracy among both temperate (Kendal's tau = 0.262, $p < 0.05$) and tropical (Kendal's tau = 0.464, $p < 0.01$) countries, but not with peak timing accuracy. Neither proportion of data missing nor proportion of population sampled was significantly associated with forecast accuracy in either the temperate or tropical regions.

Models States and Parameters

Inferred values of *S0*, R_e, R_0, *D*, and *L* for all countries and outbreaks can be found in S5 Table. Broadly, inferred states and parameters fall within realistic ranges [7–10], with values between about 50% and 90% of the population for *S0*, 1.0 and 5.3 for R_e, 1.4 and 3.1 for R_0, 2.3 to 8.4 days for *D*, and 3.8 to 7.7 years for *L*. Compared to temperate countries, countries in the tropics yielded significantly lower values of *S0*, R_0, and *L*, and significantly higher values of R_e (S14 Fig A). Within temperate regions, countries located in the northern hemisphere showed significantly higher values of both R_e and R_0 than southern hemisphere countries (S14 Fig B), and countries and seasons

reporting ILI+ data displayed significantly lower R_0 than countries reporting ARI+ data (S14 Fig C). No significant differences were observed between data types in the tropics (S14 Fig D).

Inferred Maximum and Minimum R_0 by Latitude

Neither R_{0max} nor R_{0min} varied significantly by hemisphere, but R_{0max} was significantly and negatively associated with the absolute value of latitude for both definitions of latitude used (S15 Fig A and B, and S5 Table). In other words, as the distance from the equator increased, the maximum possible *R0* tended to decrease, suggesting a weaker impact of absolute humidity at higher latitudes. That said, the relationships were weak, with Kendall's tau ranging from -0.05 to -0.10 when capital cities' latitudes were used, and from -0.10 to -0.15 when centroids were used. Such a nominal result is more likely due to the simplicity of our model or the large geographic scale at which our model is implemented, than to a true biological process.

Posterior and Forecast Visualizations

S16 Fig shows posterior model fits for five countries: Norway, Poland, Italy, Mexico, and Ecuador. These countries were chosen because they inhabit a range of latitudes and longitudes, and exhibited similar peak weeks (week 8 of 2016 for the four temperate countries, and week 17 of the same year for Ecuador). Furthermore, all four temperate countries reported ILI data. For the temperate countries, because the model was fit separately for each season, only the 2015-16 season was plotted. For Ecuador, which is located in the tropics model fitting is shown throughout the entirety of the time series leading up to the peak of interest. The mean posterior was plotted for all five model runs. As can be observed, the model was capable of closely fitting the data for a range of countries with varying locations and climates.

S17 Fig and S18 Fig show forecast trajectories over several lead weeks for the same five countries (the four temperate countries in S17 Fig, and Ecuador in S18 Fig). Both peak timing and intensity were predicted within 1 week and within 25% of observed values, respectively, by 2 weeks prior to the observed peak in Norway, Poland, and Italy, but were not both predicted accurately in Mexico until 2 weeks post-

peak. In Ecuador, as was common for countries in the tropics, neither peak timing nor peak intensity were accurately predicted until the peak occurred. Additionally, the model was unable to detect the epidemic signal, with trajectories consistently predicting decreasing rather than increasing incidence, even before the peak.

Pandemic Forecast Accuracy

In temperate regions, pandemic forecasts appear to perform slightly worse that seasonal forecasts prior to the predicted peak for both peak timing and intensity, with peak timing first exceeding 50% accuracy 2 weeks before the predicted peak, and peak intensity not exceeding 50% accuracy until the predicted peak (S19 Fig A). Given that the pandemic often did not display the clear signal and single peak typical of regular seasonal outbreaks, this finding is not surprising. Also, note that pandemic forecasting often had to be begun abruptly when out-of-season increases in influenza activity were observed.

In the tropics, forecasts of pandemic peak timing were more accurate than similar forecasts for “seasonal” influenza outbreaks several weeks before the predicted peak, and post-peak estimates of pandemic peak intensity also appeared better than analogous estimates for epidemic influenza (S19 Fig B). However, it is important to note that, because pandemic data were only available for 2 tropical countries, forecast counts are very low, reducing the certainty of these results.

REFERENCES

1. WHO Regional Office for Europe guidance for sentinel influenza surveillance in humans n.d.:144.
2. Shaman J, Karspeck A, Yang W, Tamerius J, Lipsitch M. Real-time influenza forecasts during the 2012–2013 season. Nat Commun 2013;4. doi:10.1038/ncomms3837.
3. Yang W, Cowling BJ, Lau EHY, Shaman J. Forecasting Influenza Epidemics in Hong Kong. PLOS Comput Biol 2015;11:e1004383. doi:10.1371/journal.pcbi.1004383.
4. Tofallis C. A better measure of relative prediction accuracy for model selection and model estimation. J Oper Res Soc 2015;66:1352–62. doi:10.1057/jors.2014.103.
5. Viboud C, Boëlle P-Y, Carrat F, Valleron A-J, Flahault A. Prediction of the spread of influenza epidemics by the method of analogues. Am J Epidemiol 2003;158:996–1006.
6. Yang W, Lipsitch M, Shaman J. Inference of seasonal and pandemic influenza transmission dynamics. Proc Natl Acad Sci 2015;112:2723–8. doi:10.1073/pnas.1415012112.
7. Carrat F, Flahault A. Influenza vaccine: The challenge of antigenic drift. Vaccine 2007;25:6852–62. doi:10.1016/j.vaccine.2007.07.027.
8. Truscott J, Fraser C, Hinsley W, Cauchemez S, Donnelly C, Ghani A, et al. Quantifying the transmissibility of human influenza and its seasonal variation in temperate regions. PLoS Curr 2009;1:RRN1125. doi:10.1371/currents.RRN1125.
9. White LF, Pagano M. Transmissibility of the influenza virus in the 1918 pandemic. PloS One 2008;3:e1498. doi:10.1371/journal.pone.0001498.
10. Mills CE, Robins JM, Lipsitch M. Transmissibility of 1918 pandemic influenza. Nature 2004;432:904–6. doi:10.1038/nature03063.
