# Supplementary figures and images for: Development and validation of influenza forecasting for 64 temperate and tropical countries
Source: PLoS Comput Biol. 2019 Feb 27;15(2):e1006742. doi: 10.1371/journal.pcbi.1006742 (PMC6411231; doi:10.1371/journal.pcbi.1006742)

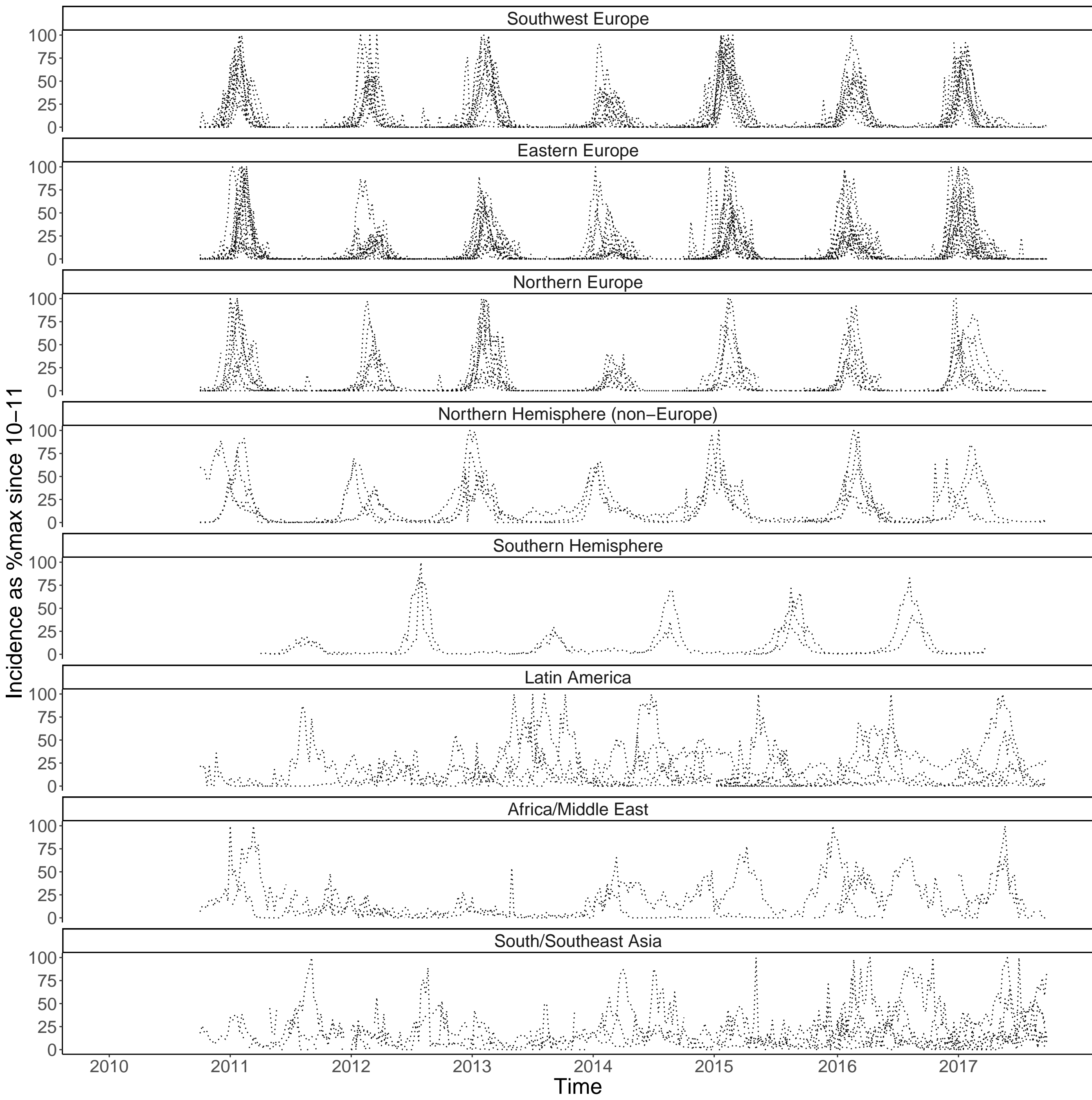

Supplement: S1 Fig — All data are divided by the maximum observed incidence in a given country since the 2010–11 season. (PDF) [file pcbi.1006742.s004.pdf]

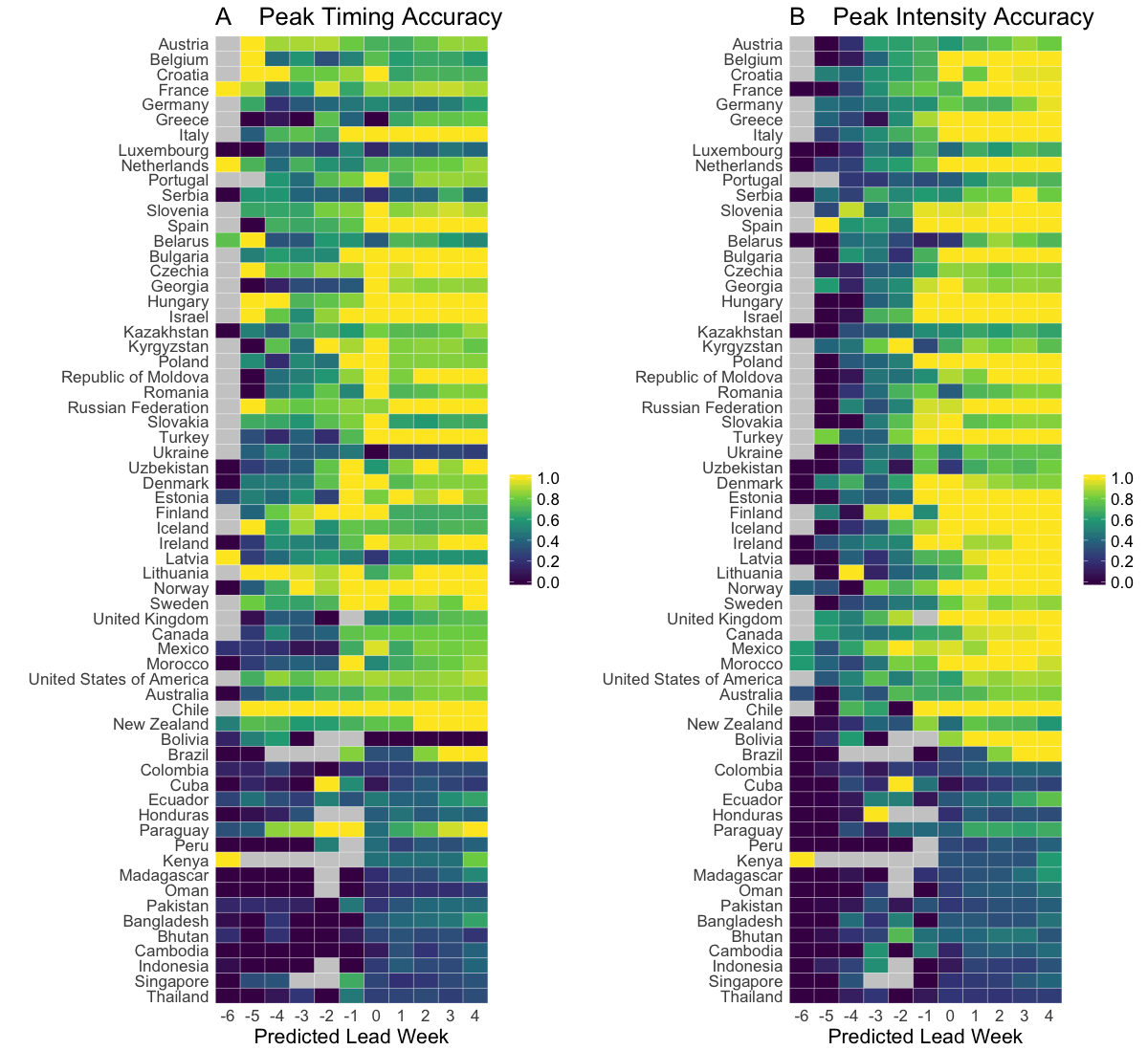

Supplement: S2 Fig — (A) Peak timing accuracy. (B) Peak intensity accuracy. NA values are represented by gray boxes. (TIF) [file pcbi.1006742.s005.tif]

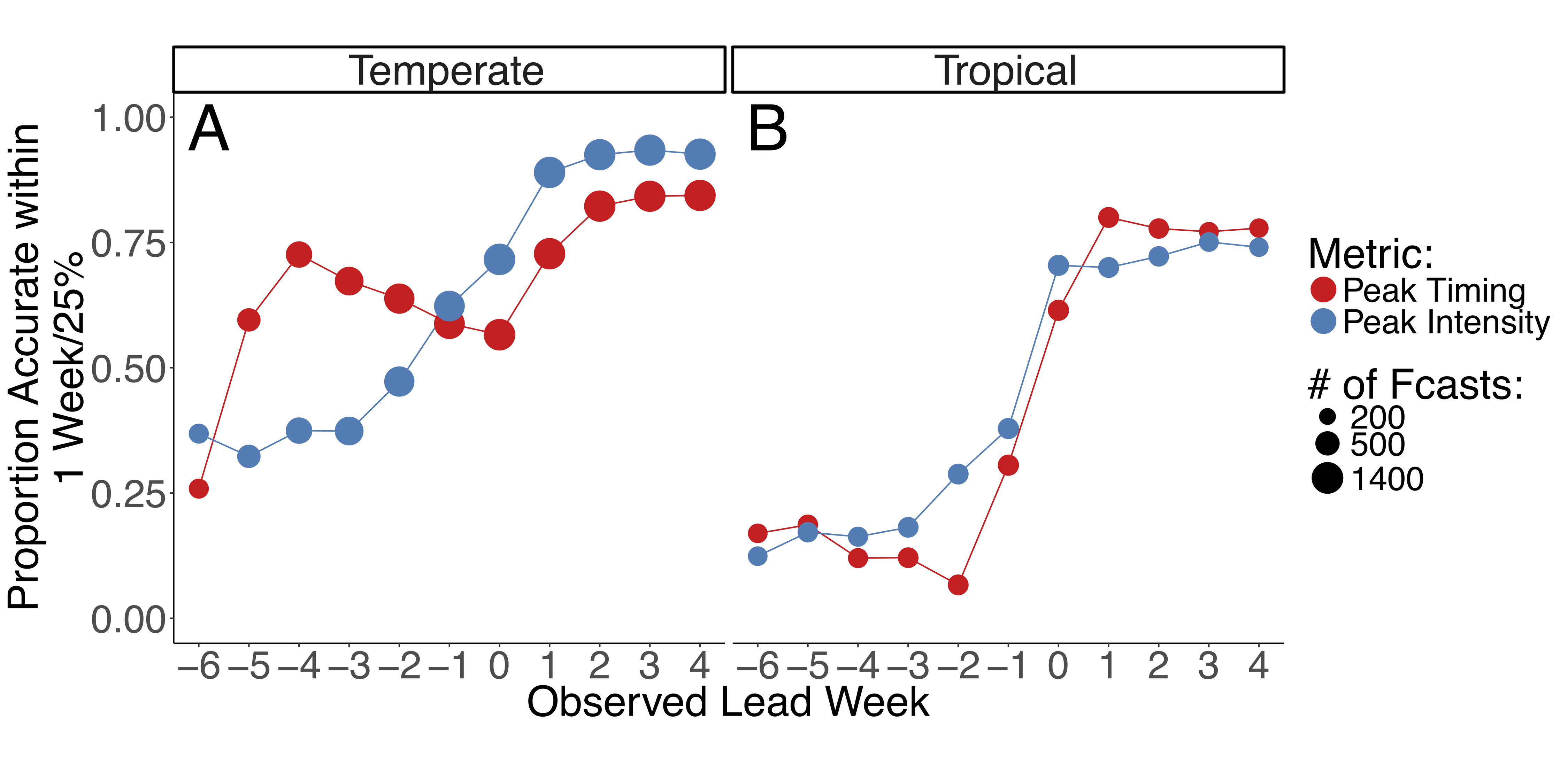

Supplement: S3 Fig — (A) Forecast accuracy in temperate regions. (B) Forecast accuracy in tropical regions. Peak timing accuracy is shown in red, and peak intensity in blue. (TIF) [file pcbi.1006742.s006.tif]

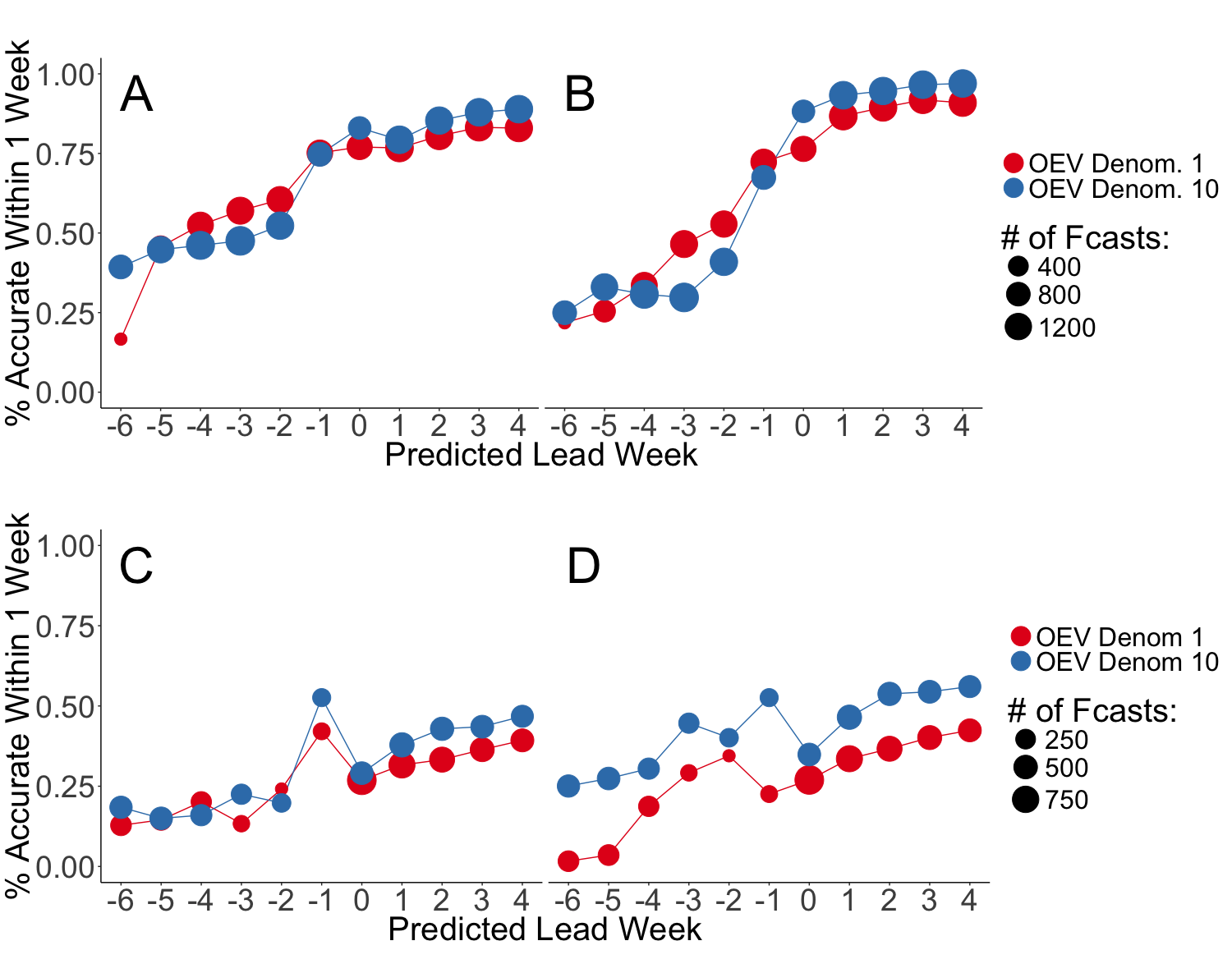

Supplement: S4 Fig — Peak timing (A and C) and intensity (B and D) accuracy for different OEV denominators in temperate (A and B) and tropical (C and D) regions. (TIF) [file pcbi.1006742.s007.tif]

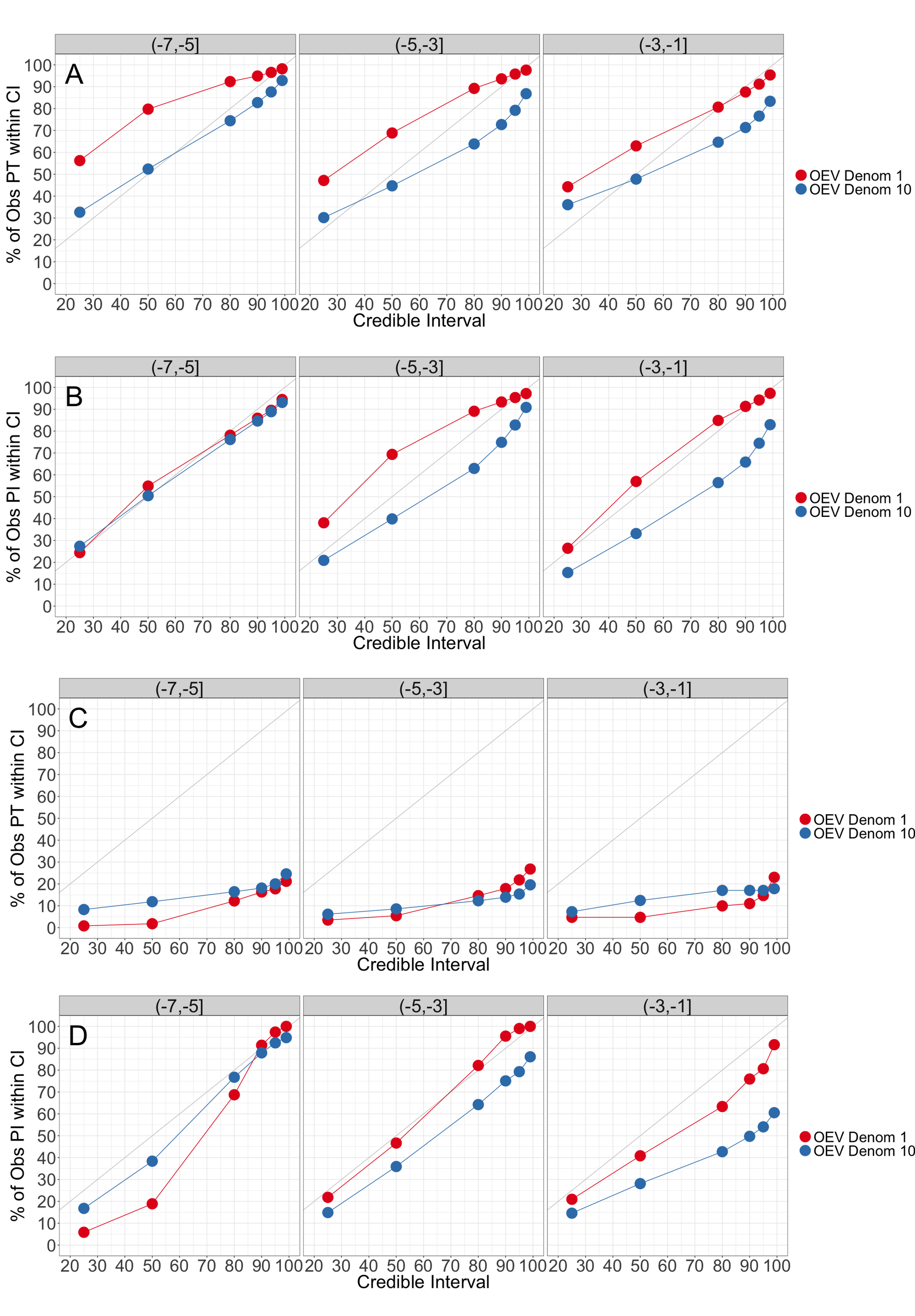

Supplement: S5 Fig — Peak timing (A and C) and intensity (B and D) calibration by OEV denominator in temperate (A and B) and tropical (C and D) regions. (TIF) [file pcbi.1006742.s008.tif]

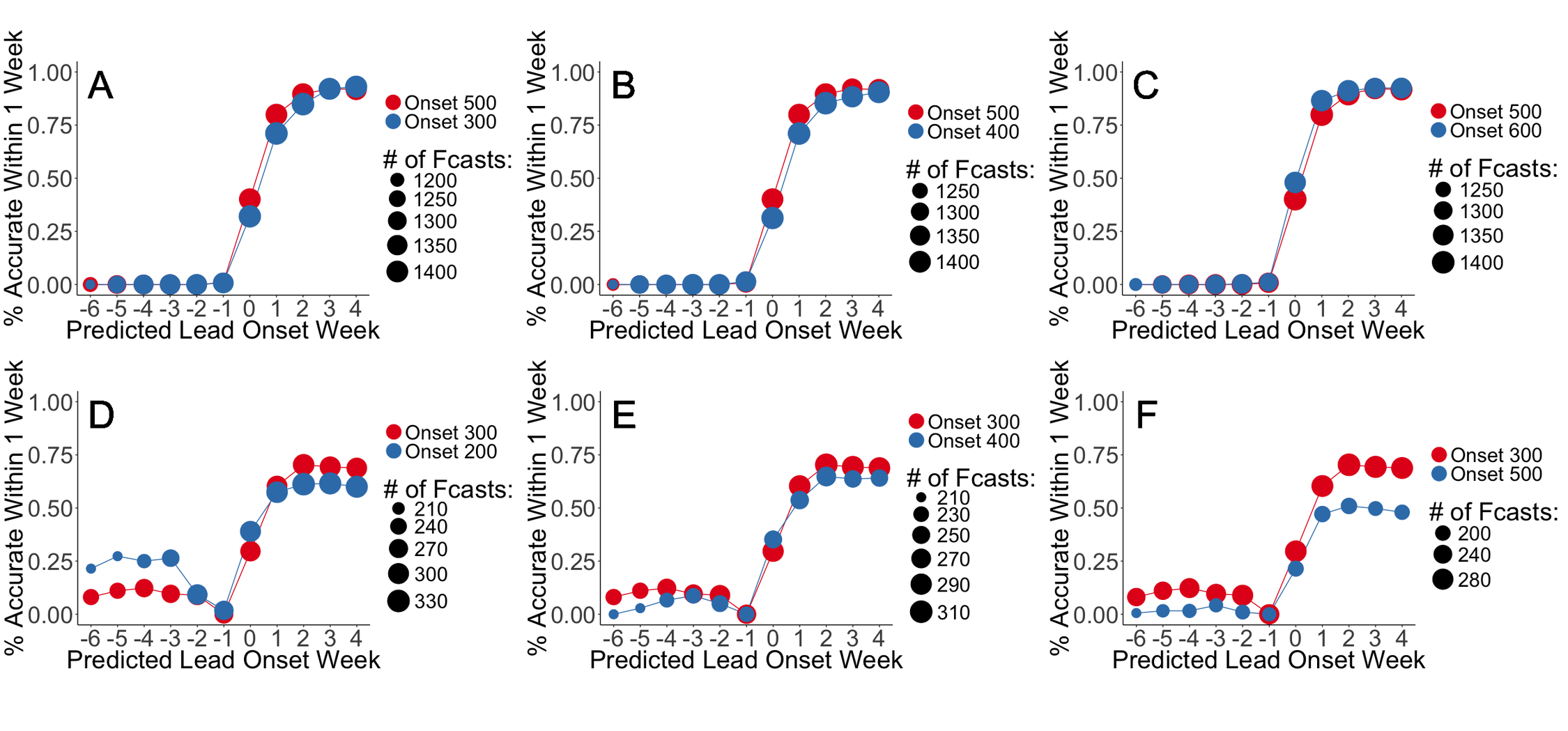

Supplement: S6 Fig — Onset timing accuracy by choice of onset value in temperate (A-C) and tropical (D-F) regions. (TIF) [file pcbi.1006742.s009.tif]

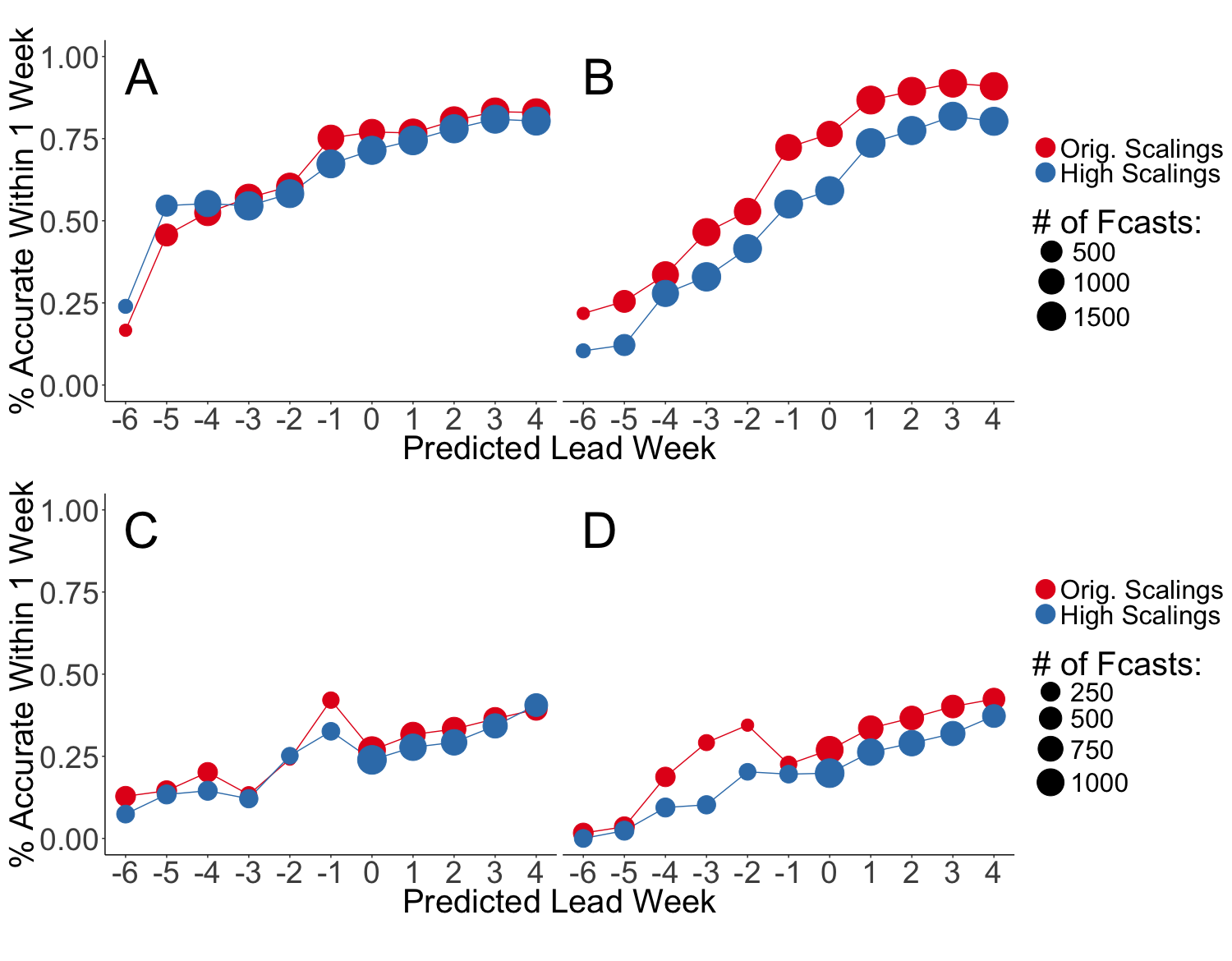

Supplement: S7 Fig — Peak timing (A and C) and intensity (B and D) accuracy by choice of scaling rule in temperate (A and B) and tropical (C and D) regions. (TIF) [file pcbi.1006742.s010.tif]

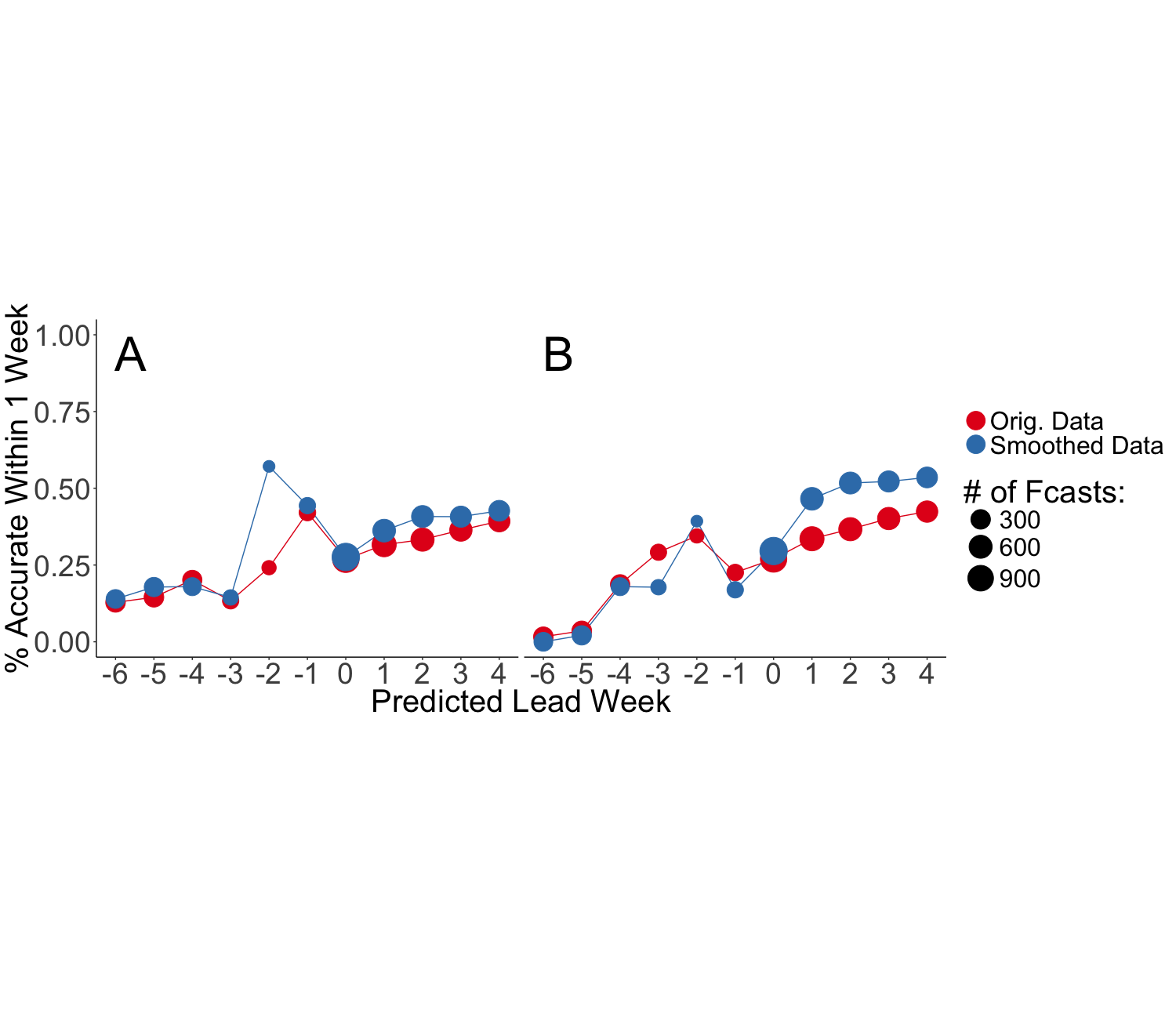

Supplement: S8 Fig — (A) Peak timing accuracy. (B) Peak intensity accuracy. (TIF) [file pcbi.1006742.s011.tif]

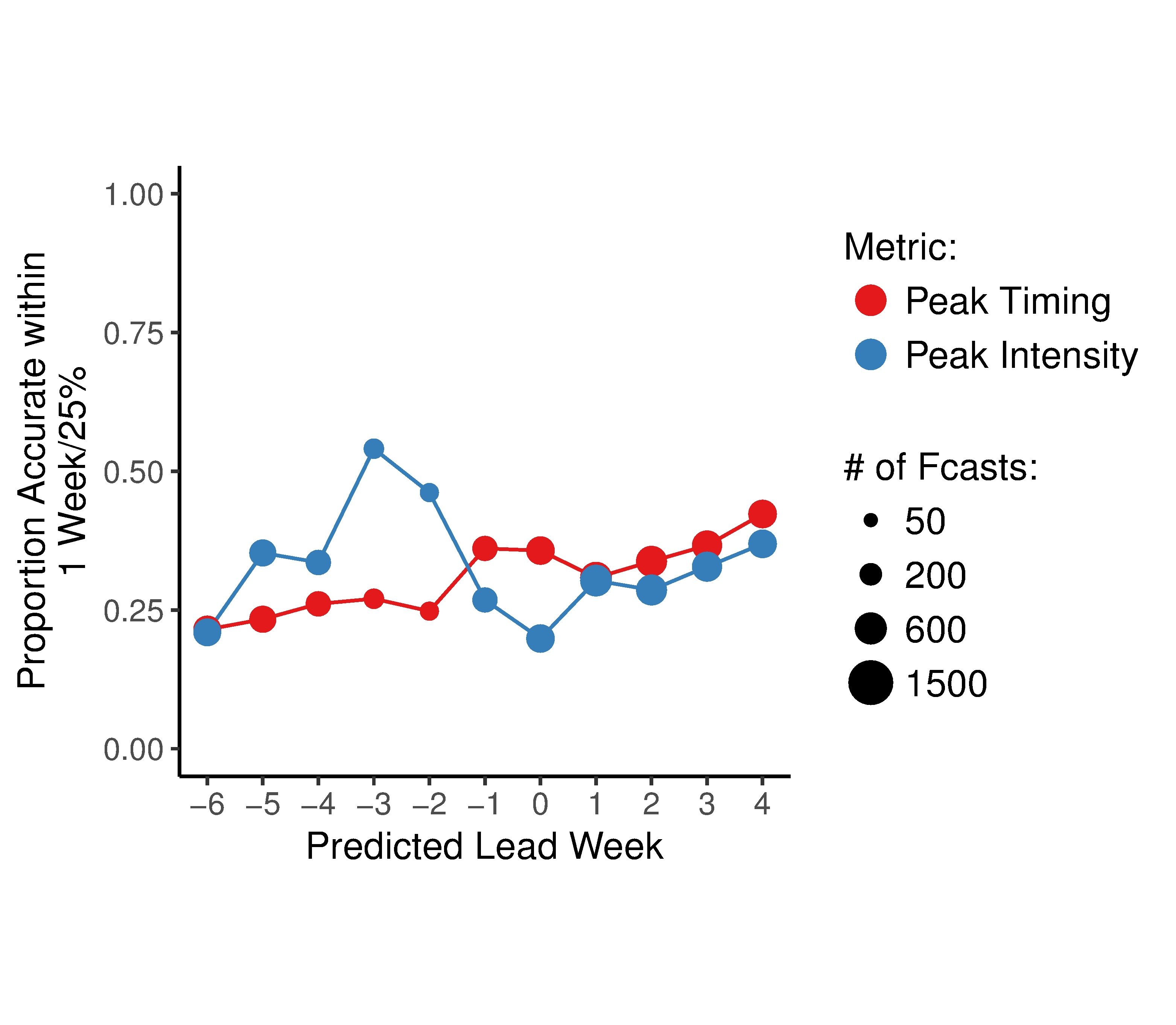

Supplement: S9 Fig — (TIF) [file pcbi.1006742.s012.tif]

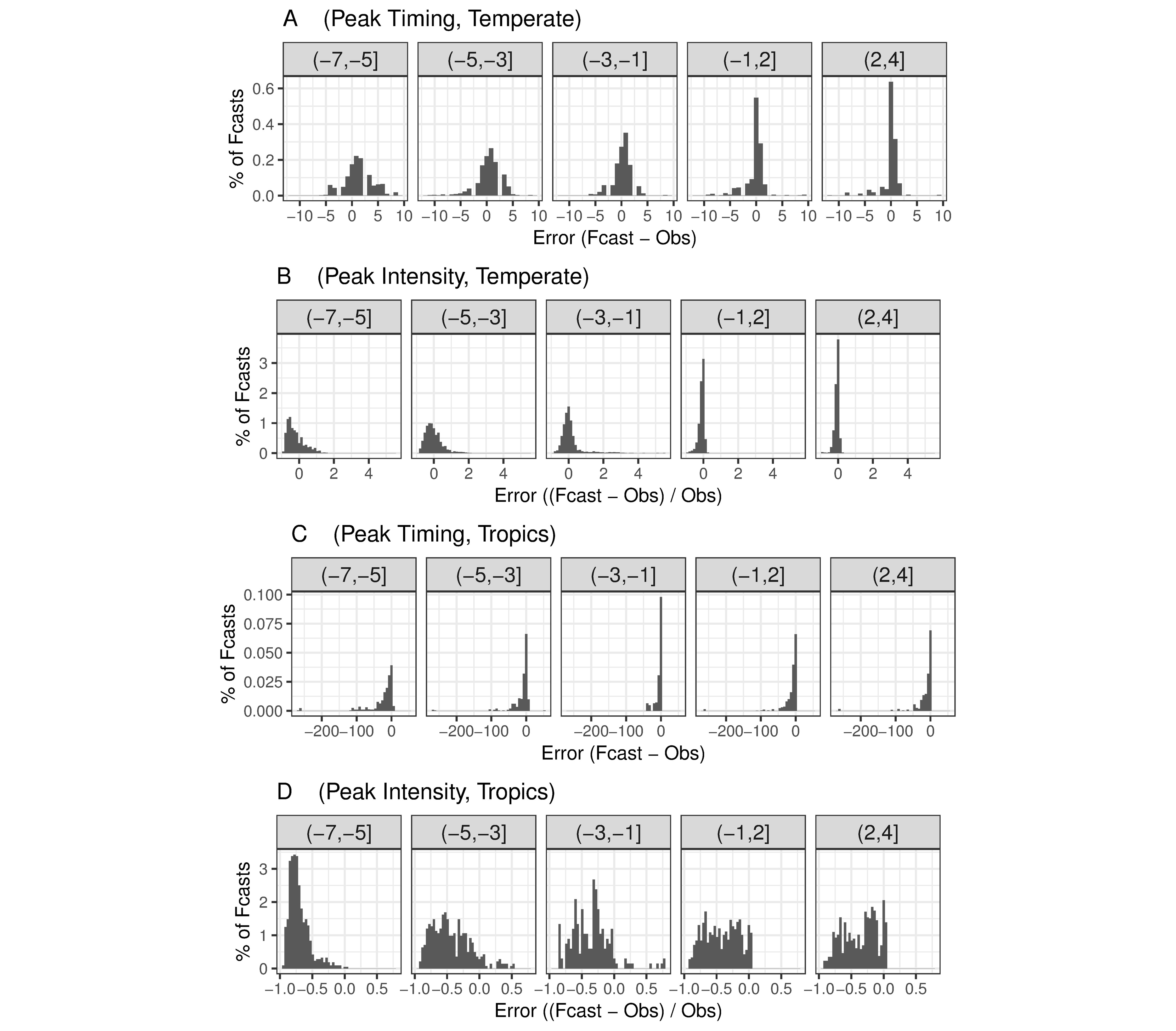

Supplement: S10 Fig — Distribution of peak timing (A and C) and intensity (B and D) errors relative to observed for temperate (A and B) and tropical (C and D) regions. To make peak intensity errors comparable between countries, errors are standardized by the observed peak intensity for a given country and season. (TIF) [file pcbi.1006742.s013.tif]

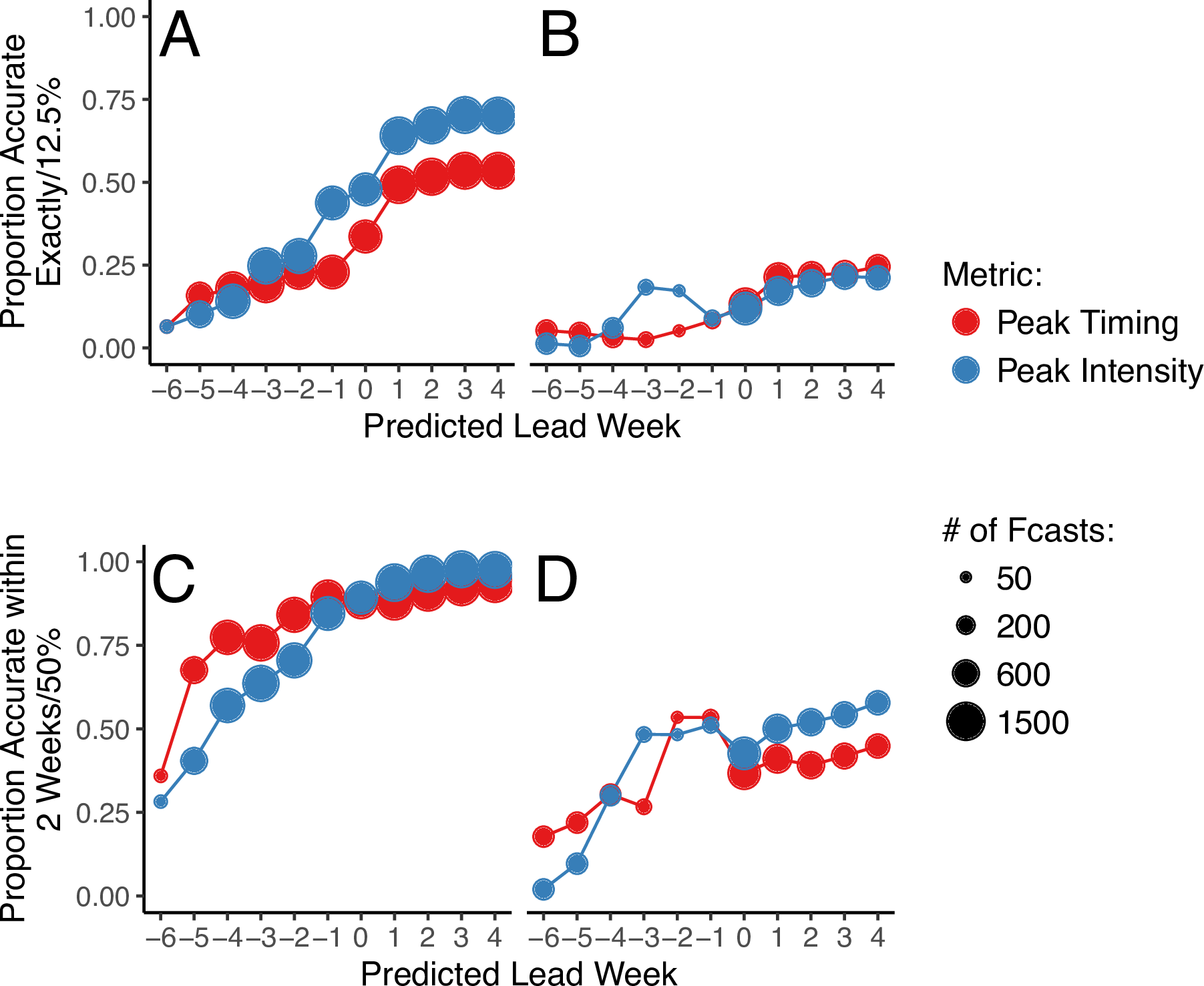

Supplement: S11 Fig — Percent of forecasts accurately predicting peak timing and intensity in temperate (A and C) and tropical (B and D) countries. (A and B) Forecasts are considered accurate if they predict peak timing exactly, and predict peak intensity within 12.5% of the observed value. (C and D) Forecasts are considered accurate when forecasts are within 2 weeks of the observed peak timing and 50% of the observed peak intensity. (TIF) [file pcbi.1006742.s014.tif]

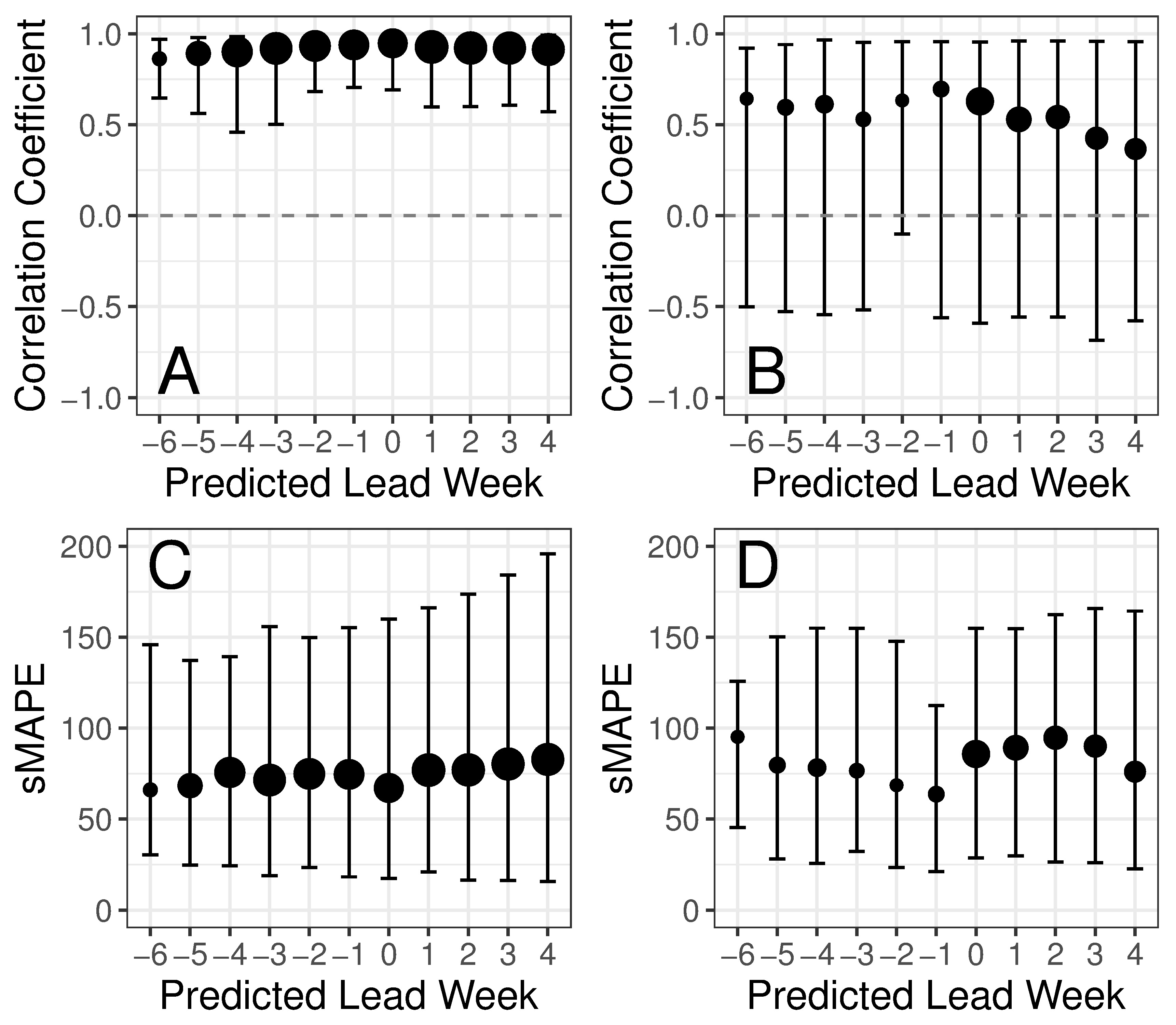

Supplement: S12 Fig — Ranges of correlation coefficients (A and B) and sMAPE (C and D) for temperate (A and C) and tropical (B and D) countries. Points represent median values, and error bars show the 95% credible interval. Point size represents the number of forecasts contributing data to the point in question. (TIF) [file pcbi.1006742.s015.tif]

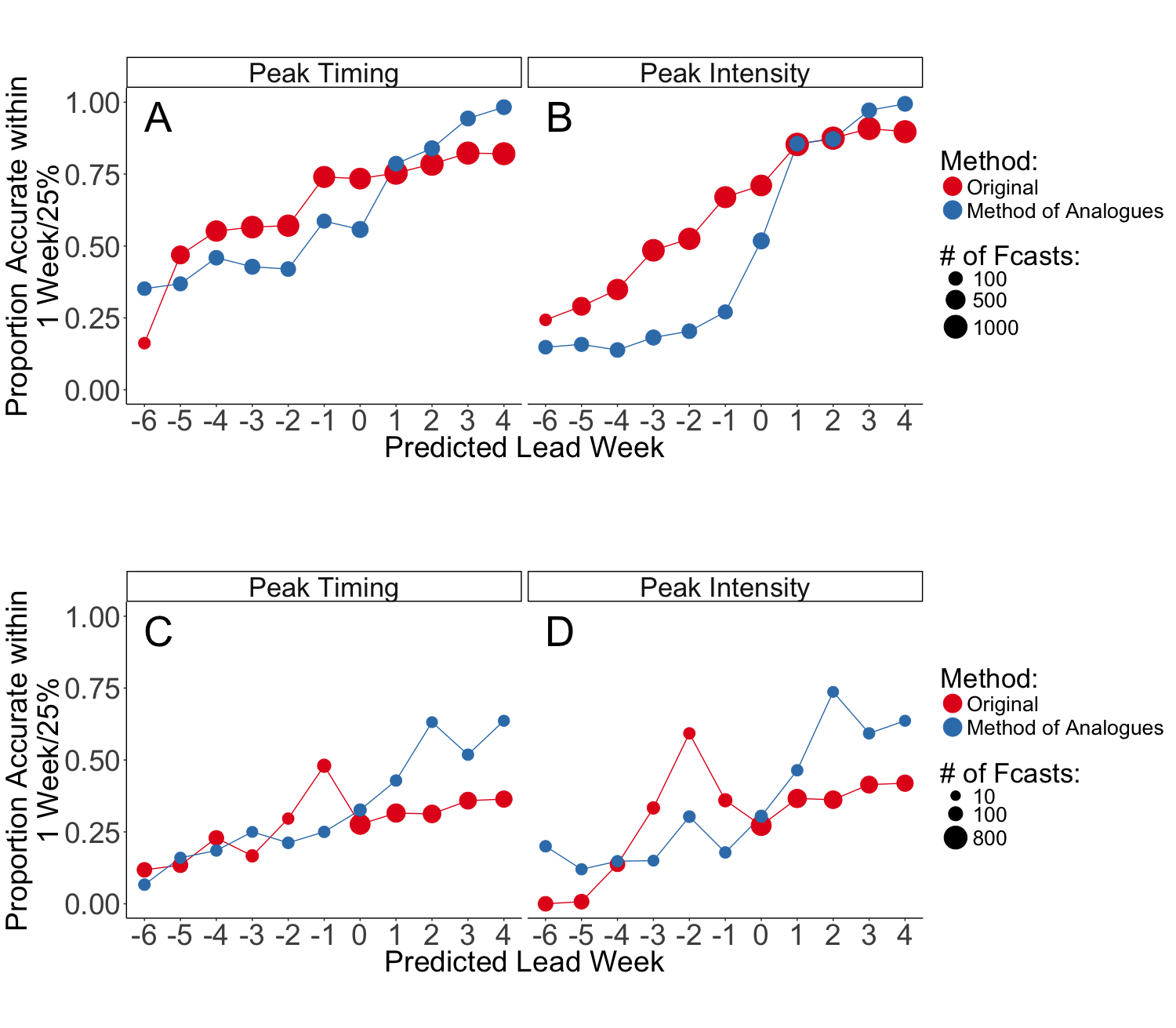

Supplement: S13 Fig — A comparison of peak timing (A and C) and peak intensity (B and D) accuracy in both temperate (A and B) and tropical (C and D) countries between the methods described in the main text (red) and the method of analogues (blue). (TIFF) [file pcbi.1006742.s016.tiff]

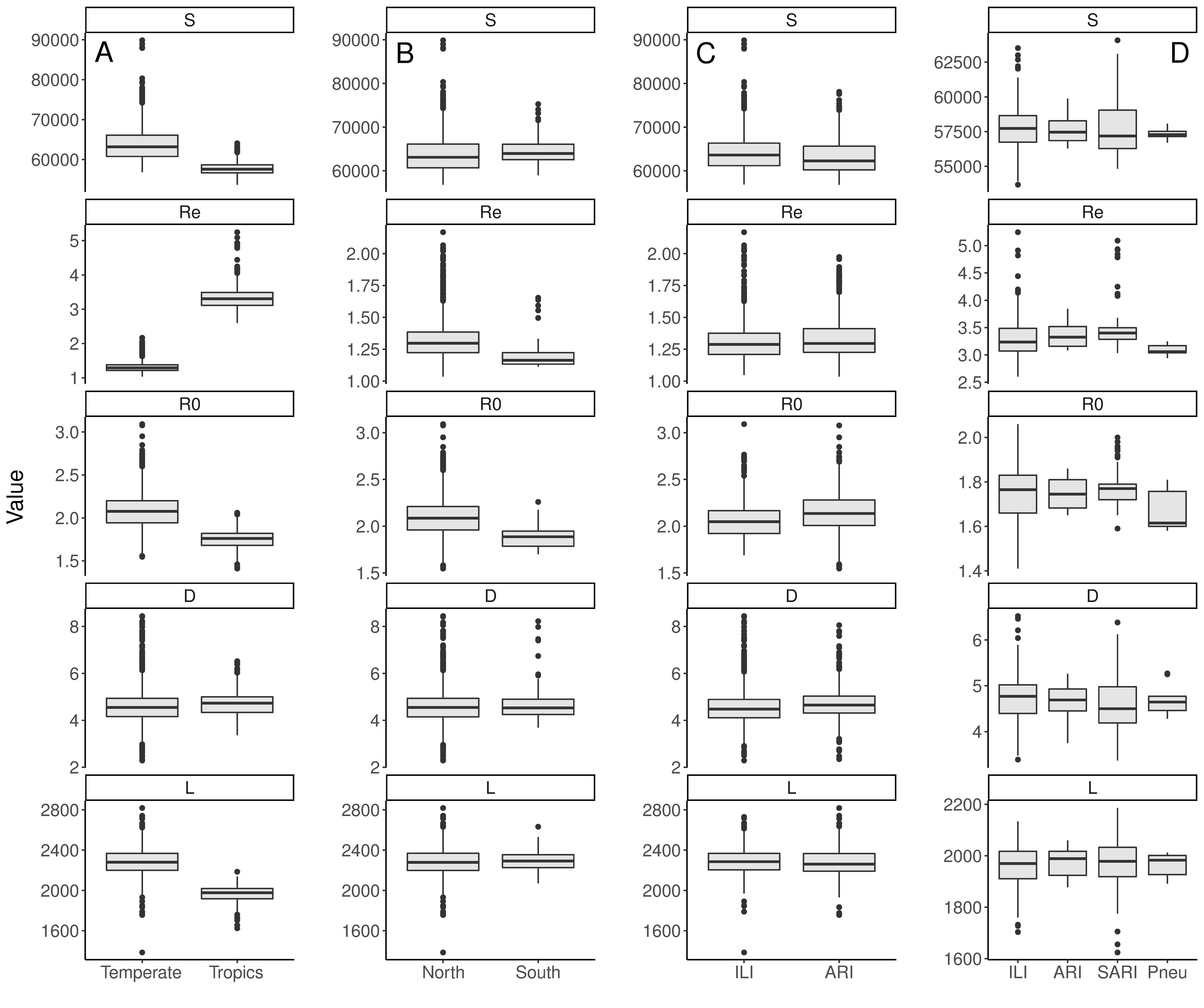

Supplement: S14 Fig — Ranges for S0, Re, R0, D, and L by temperate versus tropics designation (A), hemisphere (B), and data type separated by temperate (C) and tropical (D) regions. (TIF) [file pcbi.1006742.s017.tif]

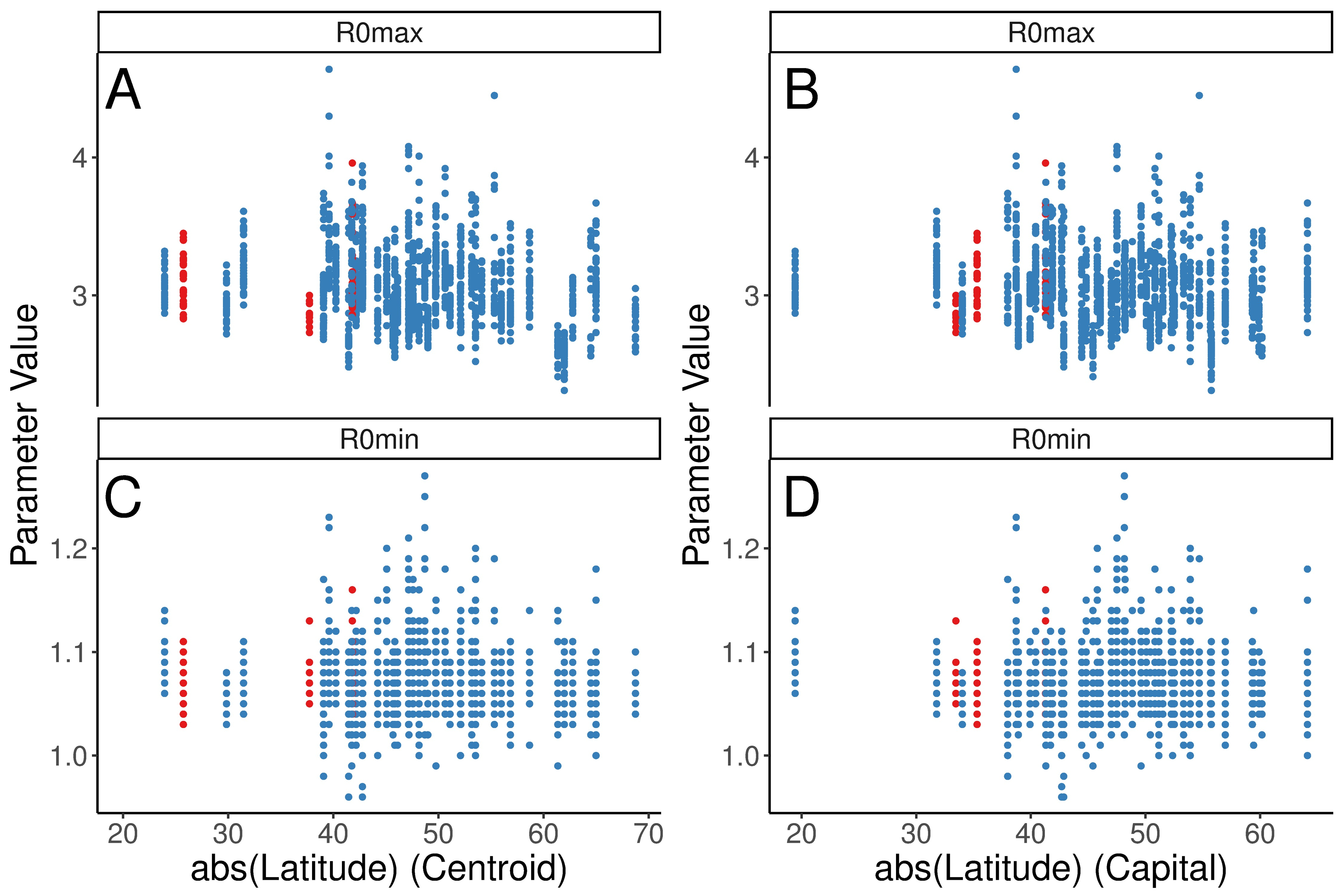

Supplement: S15 Fig — Distribution of inferred values for R0max (A and B) and R0min (C and D) by latitude (absolute value), defined as the latitude at the country’s centroid (A and C) or the latitude of the country’s capital (B and D). Values derived from temperate countries are shown in blue, and values from countries in the tropics are in red. (TIF) [file pcbi.1006742.s018.tif]

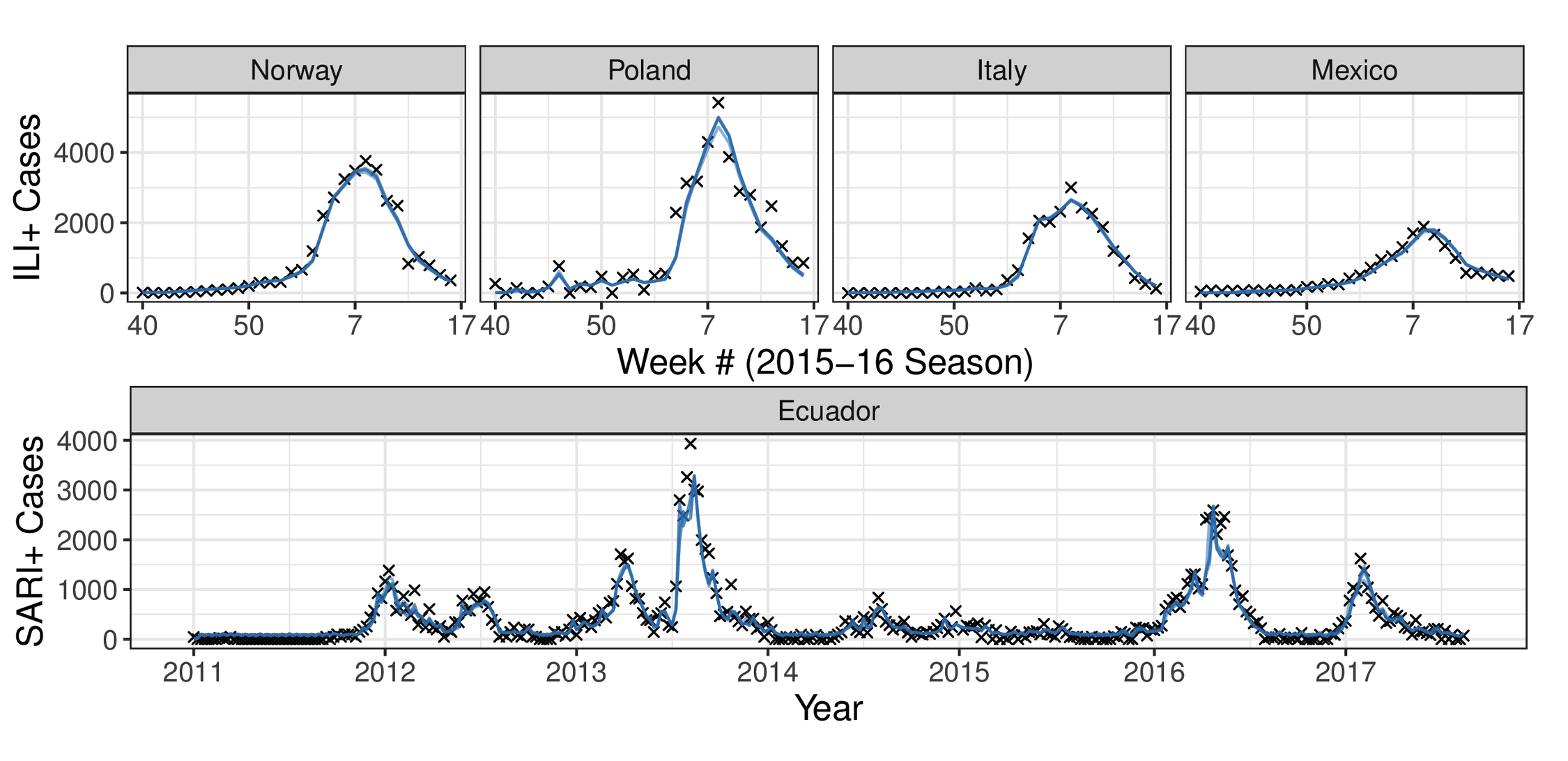

Supplement: S16 Fig — Mean posterior fit for 5 models runs of 300 ensemble members each for Norway, Poland, Italy, Mexico, and Ecuador. Fit is plotted for the 2015–16 season for the temperate countries, and for the entire duration of the available data for Ecuador. Observed data are plotted as black x’s, while the posterior model fit is plotted in blue. (TIF) [file pcbi.1006742.s019.tif]

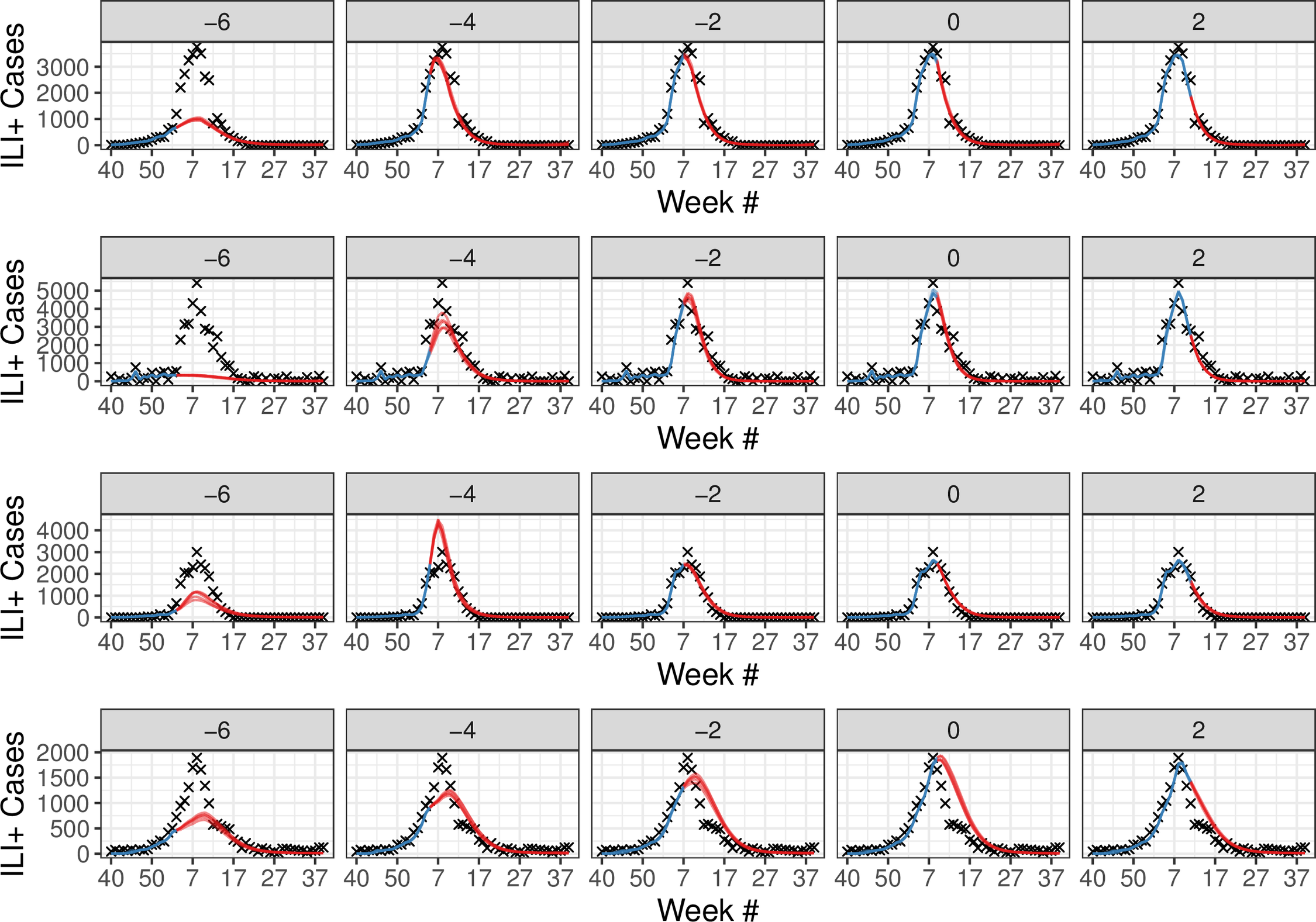

Supplement: S17 Fig — Forecast trajectories for Norway, Poland, Italy, and Mexico for the 2015–16 season. Forecasts are presented starting 6 weeks prior to the observed week through 2 weeks after the peak. Black x’s represent observed data, blue lines show model incidence during the training period, and red lines represent forecast trajectory. For each forecast, the 5 runs are shown separately. (TIF) [file pcbi.1006742.s020.tif]

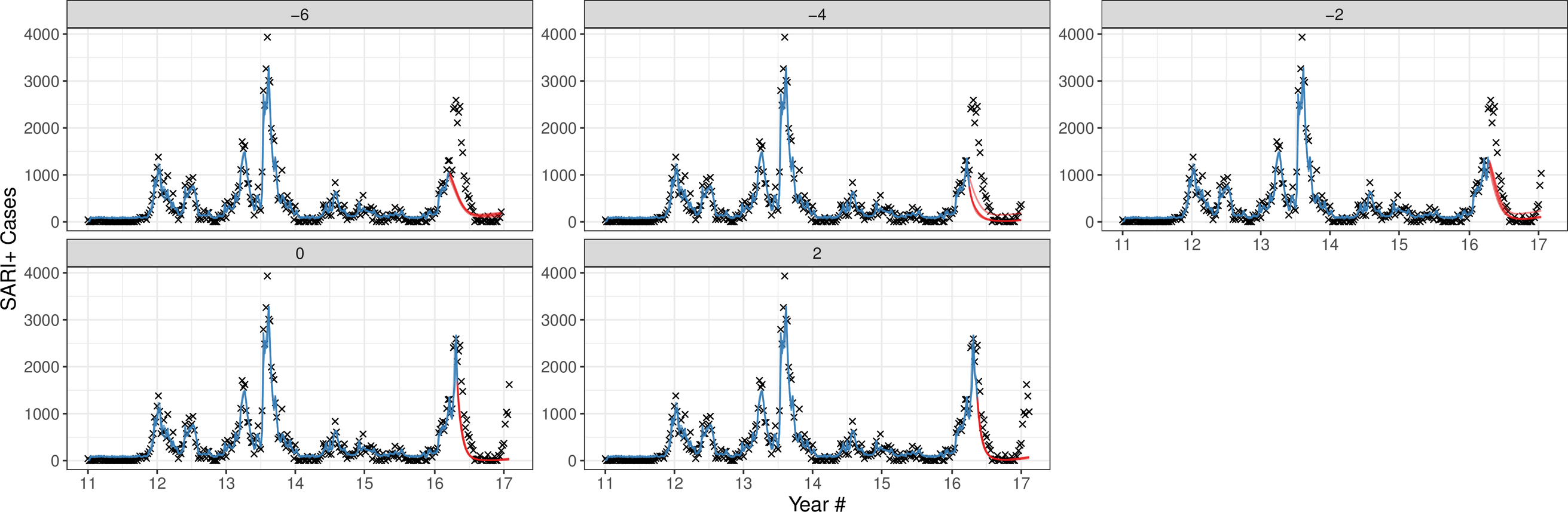

Supplement: S18 Fig — Forecast trajectory for the fifth recorded outbreak in Ecuador in our dataset. Forecasts are presented as in S17 Fig. Because tropical forecasts were generated by fitting the model to the observations continuously, rather than by season, model fitting for all data prior to the fifth outbreak in Ecuador is shown. (TIF) [file pcbi.1006742.s021.tif]

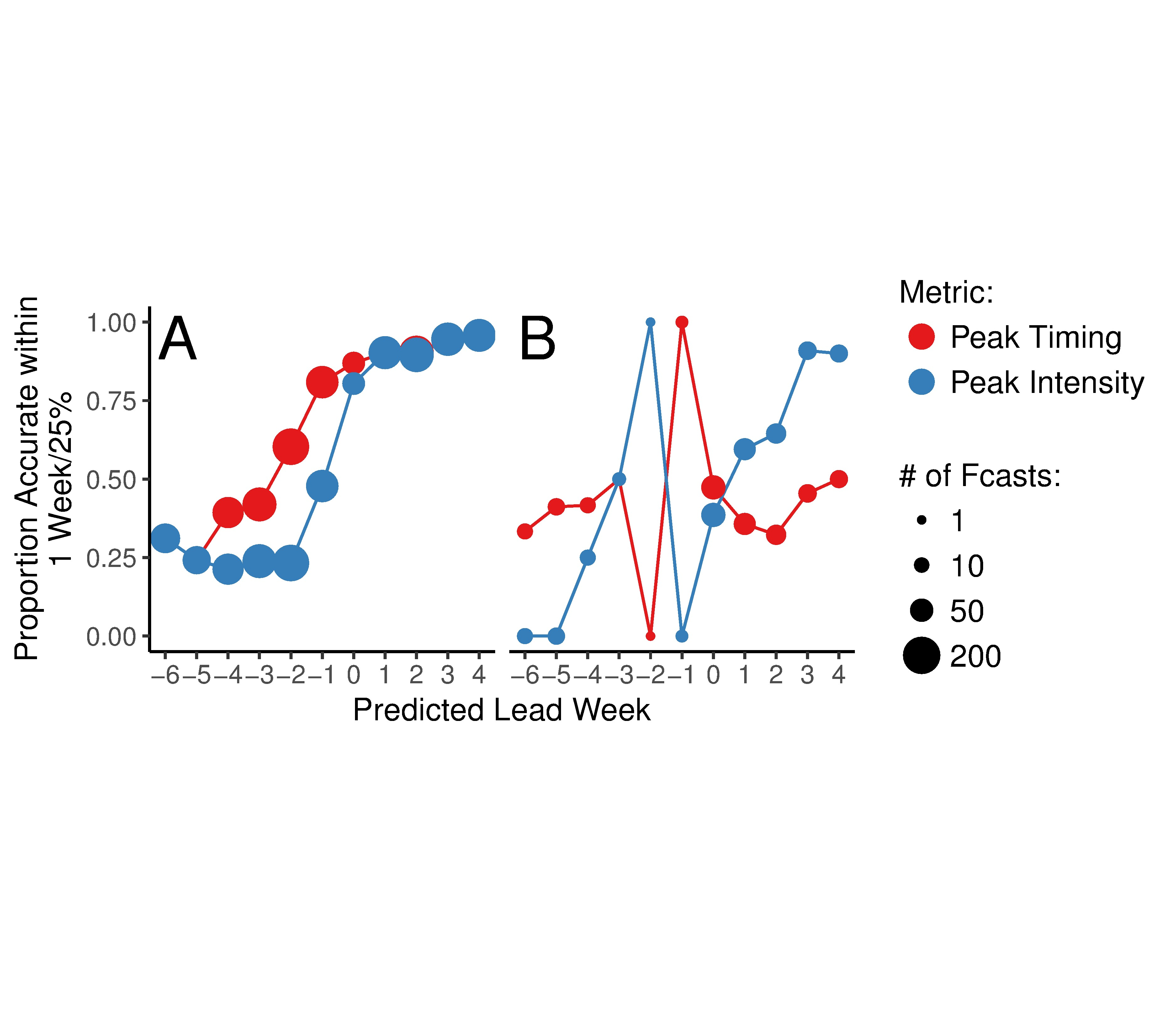

Supplement: S19 Fig — Peak timing (red) and intensity (blue) accuracy for forecasts of the 2009 pandemic in temperate (A) and tropical (B) countries. (TIF) [file pcbi.1006742.s022.tif]
