# Supplementary material for: Development and validation of influenza forecasting for 64 temperate and tropical countries
Source: PLoS Comput Biol. 2019 Feb 27;15(2):e1006742. doi: 10.1371/journal.pcbi.1006742 (PMC6411231; doi:10.1371/journal.pcbi.1006742)
Supplement: S1 Table — (PDF) [file pcbi.1006742.s023.pdf]

**Table S1: Countries used for retrospective forecasting, by region, data type, and scaling.**

| Country | Hemisphere | Region | Data Type | Scaling |
|---|---|---|---|---|
| Austria | North | Southwest Europe | ILI | 14 |
| Belgium | North | Southwest Europe | ILI | 8.5 |
| Croatia | North | Southwest Europe | ILI | 1.4 |
| France | North | Southwest Europe | ARI/ILI | 1.3/0.03 |
| Germany | North | Southwest Europe | ARI | 0.25 |
| Greece | North | Southwest Europe | ILI | 4 |
| Italy | North | Southwest Europe | ILI | 1 |
| Luxembourg | North | Southwest Europe | ARI | 32 |
| Netherlands | North | Southwest Europe | ILI | 31 |
| Portugal | North | Southwest Europe | ILI | 245 |
| Serbia | North | Southwest Europe | ILI | 0.5 |
| Slovenia | North | Southwest Europe | ARI | 5.8 |
| Spain | North | Southwest Europe | ILI | 2 |
| Belarus | North | Eastern Europe | ARI | 0.2 |
| Bulgaria | North | Eastern Europe | ARI | 1.25 |
| Czechia | North | Eastern Europe | ILI | 0.8 |
| Georgia | North | Eastern Europe | ILI | 15 |
| Hungary | North | Eastern Europe | ILI | 1 |
| Israel | North | Eastern Europe | ILI | 3.5 |
| Kazakhstan | North | Eastern Europe | ARI | 0.35 |
| Kyrgyzstan | North | Eastern Europe | ARI | 0.63 |
| Poland | North | Eastern Europe | ILI | 1.3 |
| Republic of Moldova | North | Eastern Europe | ARI | 2.25 |
| Romania | North | Eastern Europe | ILI | 39 |
| Russian Federation | North | Eastern Europe | ARI | 0.02 |
| Slovakia | North | Eastern Europe | ILI | 0.57 |
| Turkey | North | Eastern Europe | ILI | 3.5 |
| Ukraine | North | Eastern Europe | ARI | 0.03 |
| Uzbekistan | North | Eastern Europe | ARI | 68 |
| Denmark | North | Northern Europe | ILI | 18 |
| Estonia | North | Northern Europe | ARI | 1.5 |
| Finland | North | Northern Europe | ILI | 14 |
| Iceland | North | Northern Europe | ILI | 57 |
| Ireland | North | Northern Europe | ILI | 45 |
| Latvia | North | Northern Europe | ARI | 4.4 |
| Lithuania | North | Northern Europe | ILI | 1.3 |
| Norway | North | Northern Europe | ILI | 1.65 |
| Sweden | North | Northern Europe | ILI | 132 |
| United Kingdom | North | Northern Europe | ARI | 1.8 |
| Canada | North | Northern Hemisphere (non-Europe) | ILI Rate | 182 |
| Mexico | North | Northern Hemisphere (non-Europe) | ARI | 0.004 |
| Morocco | North | Northern Hemisphere (non-Europe) | ILI | 0.78 |
| United States of America | North | Northern Hemisphere (non-Europe) | ILI | 0.27 |

| | | | | |
|---|---|---|---|---|
| Australia | South | Southern Hemisphere | ILI | 32 |
| Chile | South | Southern Hemisphere | ILI | 31 |
| New Zealand | South | Southern Hemisphere | ILI | 16 |
| Brazil | Tropics | Latin America | SARI | 6 |
| Bolivia | Tropics | Latin America | ARI | 0.03 |
| Colombia | Tropics | Latin America | ARI | 0.075 |
| Cuba | Tropics | Latin America | ILI | 127 |
| Ecuador | Tropics | Latin America | SARI | 48 |
| Honduras | Tropics | Latin America | Pneumonia | 2 |
| Paraguay | Tropics | Latin America | ILI | 3.5 |
| Peru | Tropics | Latin America | SARI | 374 |
| Kenya | Tropics | Africa/Middle East | ILI | 10 |
| Madagascar | Tropics | Africa/Middle East | ILI | 6.6 |
| Oman | Tropics | Africa/Middle East | SARI | 6.4 |
| Pakistan | Tropics | Africa/Middle East | ILI | 93 |
| Bangladesh | Tropics | South/Southeast Asia | SARI | 48 |
| Bhutan | Tropics | South/Southeast Asia | ILI | 12 |
| Cambodia | Tropics | South/Southeast Asia | SARI | 90 |
| Indonesia | Tropics | South/Southeast Asia | ILI | 26 |
| Singapore | Tropics | South/Southeast Asia | ILI | 4.7 |
| Thailand | Tropics | South/Southeast Asia | ILI | 0.04 |
