# Supplementary material for: Development and validation of influenza forecasting for 64 temperate and tropical countries
Source: PLoS Comput Biol. 2019 Feb 27;15(2):e1006742. doi: 10.1371/journal.pcbi.1006742 (PMC6411231; doi:10.1371/journal.pcbi.1006742)
Supplement: S2 Table — (PDF) [file pcbi.1006742.s024.pdf]

Table S2: Countries and seasons used for retrospective forecasting.

Country	2009 pdm	10-11	11-12	12-13	13-14	14-15	15-16	16-17
Austria		x	x	x	x	x	x	x
Belgium	x	x	x	x	x	x	x	x
Croatia	x	x	x	x	x	x	x	x
France				x	x	x	x	x
Germany	x	x	x	x	x	x	x	x
Greece	x	x	x	x	x	x		x
Italy		x	x	x	x	x	x	x
Luxembourg	x	x	x	x	x	x	x	x
Netherlands	x	x	x	x	x	x	x	x
Portugal		x	x	x	x	x	x	x
Serbia	x	x		x	x	x	x	
Slovenia	x	x	x	x	x	x	x	x
Spain	x	x	x	x	x	x	x	x
Belarus		x	x	x	x	x	x	x
Bulgaria	x	x	x	x	x	x	x	x
Czechia	x	x	x	x		x	x	x
Georgia	x	x	x	x	x	x	x	x
Hungary	x	x	x	x	x	x	x	x
Israel	x	x	x	x	x	x	x	x
Kazakhstan		x	x	x	x	x	x	x
Kyrgyzstan	x	x	x	x		x	x	x
Poland	x	x		x	x	x	x	x
Republic of Moldova	x	x	x	x	x	x	x	x
Romania	x	x	x	x	x	x	x	x
Russian Federation	x	x	x	x	x	x	x	x
Slovakia	x	x	x	x	x	x	x	x
Turkey	x	x	x	x	x	x	x	x
Ukraine	x	x	x	x	x	x	x	x
Uzbekistan				x	x	x	x	x
Denmark	x	x	x	x	x	x	x	x
Estonia	x	x	x	x	x	x	x	x
Finland				x	x	x	x	x
Iceland		x	x	x	x	x	x	x
Ireland	x	x	x	x	x	x	x	x
Latvia	x	x	x	x	x	x	x	x
Lithuania		x		x		x	x	x
Norway	x	x	x	x		x	x	x
Sweden	x	x	x	x	x			
United Kingdom	x	x	x	x	x			
Canada	x	x	x	x	x		x	
Mexico			x	x	x	x	x	x
Morocco	x	x	x	x		x	x	x
United States of America	x	x	x	x	x	x	x	x
Australia			x	x	x	x	x	x
Chile							x	x
New Zealand			x	x	x	x	x	
