# Supplementary material for: Development and validation of influenza forecasting for 64 temperate and tropical countries
Source: PLoS Comput Biol. 2019 Feb 27;15(2):e1006742. doi: 10.1371/journal.pcbi.1006742 (PMC6411231; doi:10.1371/journal.pcbi.1006742)
Supplement: S3 Table — Cells shaded in green indicate improved forecast accuracy over the reference level, while cells shaded in red indicate reduced accuracy. (PDF) [file pcbi.1006742.s025.pdf]

**Table S3. Peak timing and intensity accuracy overall, before the predicted peak, and at or after the predicted peak in temperate regions by hemisphere, season, region, data type, and scaling.** Cells shaded in green indicate improved forecast accuracy over the reference level, while cells shaded in red indicate reduced accuracy.

| Variable | | Peak Timing aOR (95% CI) | | | Peak Intensity aOR (95% CI) | | |
|---|---|---|---|---|---|---|---|
| | | Overall | Before Peak | After Peak | Overall | Before Peak | After Peak |
| Hemisphere | Southern | 2.125 (0.421, 10.684) | 1.428 (0.562, 3.599) | 1.134 (0.382, 3.384) | 0.693 (0.269, 1.765) | 0.940 (0.432, 2.052) | 0.436 (0.108, 1.725) |
| Season | 2016-17 | 1.00 (ref) | 1.00 (ref) | 1.00 (ref) | 1.00 (ref) | 1.00 (ref) | 1.00 (ref) |
| | 2015-16 | 1.252 (0.603, 2.624) | 1.105 (0.552, 2.211) | 0.670 (0.269, 1.693) | 0.795 (0.450, 1.401) | 0.764 (0.437, 1.335) | 0.761 (0.223, 2.570) |
| | 2014-15 | 0.997 (0.481, 2.079) | 1.004 (0.470, 2.127) | 1.274 (0.434, 3.736) | 1.071 (0.630, 1.836) | 0.958 (0.543, 1.678) | 1.289 (0.327, 4.810) |
| | 2013-14 | 0.963 (0.441, 2.134) | 0.752 (0.347, 1.636) | 0.781 (0.304, 2.014) | 1.915 (0.991, 3.750) | 1.841 (0.969, 3.493) | Inf (0.292, Inf) |
| | 2012-13 | 1.047 (0.499, 2.200) | 1.177 (0.570, 2.437) | 0.985 (0.365, 2.668) | 1.115 (0.638, 1.950) | 1.145 (0.640, 2.030) | 0.701 (0.214, 2.330) |
| | 2011-12 | 0.933 (0.433, 2.013) | 0.911 (0.419, 1.980) | 1.141 (0.391, 3.332) | 1.433 (0.869, 2.341) | 1.583 (0.925, 2.678) | 1.431 (0.324, 6.128) |
| | 2010-11 | 1.540 (0.726, 3.306) | 1.333 (0.664, 2.665) | 0.783 (0.298, 2.062) | 1.033 (0.619, 1.726) | 0.911 (0.526, 1.577) | 1.610 (0.383, 7.018) |
| Region | SW Europe | 1.00 (ref) | 1.00 (ref) | 1.00 (ref) | 1.00 (ref) | 1.00 (ref) | 1.00 (ref) |
| | E Europe | 1.216 (0.737, 1.999) | 1.045 (0.645, 1.690) | 2.068 (1.095, 3.889) | 0.744 (0.519, 1.067) | 0.770 (0.527, 1.126) | 0.962 (0.431, 2.126) |
| | N Europe | 1.284 (0.691, 2.401) | 1.286 (0.714, 2.324) | 1.515 (0.718, 3.199) | 1.114 (0.775, 1.605) | 1.039 (0.674, 1.609) | Inf (1.034, Inf) |
| | N Hem (non-Europe) | 0.807 (0.380, 1.736) | 0.844 (0.403, 1.750) | 2.036 (0.726, 5.745) | 1.713 (0.835, 3.453) | 1.686 (0.839, 3.356) | 2.700 (0.458, Inf) |
| | S Hem | 2.331 (0.471, 11.672) | 1.475 (0.556, 3.955) | 1.716 (0.548, 5.377) | 0.656 (0.247, 1.743) | 0.905 (0.403, 2.055) | 0.548 (0.122, 2.386) |
| Data Type | ARI+ | 0.639 (0.421, 0.968) | 0.645 (0.428, 0.973) | 0.567 (0.334, 0.965) | 0.832 (0.606, 1.132) | 0.776 (0.562, 1.084) | 0.741 (0.325, 1.721) |
| Scaling | (2, 10] | 1.00 (ref) | 1.00 (ref) | 1.00 (ref) | 1.00 (ref) | 1.00 (ref) | 1.00 (ref) |
| | (0, 0.5] | 0.771 (0.399, 1.488) | 0.920 (0.473, 1.783) | 0.420 (0.181, 0.982) | 0.633 (0.397, 1.007) | 0.846 (0.498, 1.421) | 0.170 (0.051, 0.568) |
| | (0.5, 1] | 1.361 (0.636, 2.941) | 1.452 (0.701, 3.008) | 1.758 (0.618, 5.075) | 0.862 (0.524, 1.412) | 1.044 (0.599, 1.818) | 0.374 (0.100, 1.511) |
| | (1, 2] | 1.360 (0.705, 2.612) | 1.379 (0.704, 2.686) | 1.815 (0.641, 5.115) | 0.957 (0.648, 1.409) | 1.058 (0.631, 1.765) | 2.387 (0.412, 13.290) |
| | (10, 20] | 1.869 (0.729, 4.785) | 1.441 (0.667, 3.086) | 0.799 (0.286, 2.244) | 0.681 (0.401, 1.153) | 1.115 (0.626, 1.990) | 0.145 (0.038, 0.571) |
| | (20, 100] | 0.863 (0.449, 1.650) | 0.932 (0.483, 1.801) | 0.667 (0.266, 1.674) | 0.609 (0.374, 1.004) | 0.873 (0.519, 1.463) | 0.229 (0.066, 0.801) |
| | (100, 500] | 1.494 (0.516, 4.120) | 1.227 (0.520, 2.857) | 0.956 (0.274, 3.336) | 0.479 (0.250, 0.909) | 0.604 (0.276, 1.345) | 0.147 (0.033, 0.646) |
