# Supplementary material for: Development and validation of influenza forecasting for 64 temperate and tropical countries
Source: PLoS Comput Biol. 2019 Feb 27;15(2):e1006742. doi: 10.1371/journal.pcbi.1006742 (PMC6411231; doi:10.1371/journal.pcbi.1006742)
Supplement: S4 Table — Cells shaded in green indicate improved forecast accuracy over the reference level, while cells shaded in red indicate reduced accuracy. (PDF) [file pcbi.1006742.s026.pdf]

Table S4. Peak timing and intensity accuracy at or after the predicted peak in the tropics by region, data type, and scaling. Cells shaded in green indicate improved forecast accuracy over the reference level, while cells shaded in red indicate reduced accuracy.

Variable		Peak Timing aOR (95% CI)	Peak Intensity aOR (95% CI)
Region	Latin America	1.00 (ref)	1.00 (ref)
	N Africa/Middle East	0.730 (0.420, 1.279)	0.751 (0.412, 1.374)
	SE Asia	0.722 (0.491, 1.057)	0.799 (0.425, 1.516)
Data Type	ILI+	1.00 (ref)	1.00 (ref)
	ARI+	0.822 (0.505, 1.341)	1.772 (0.726, 4.335)
	SARI+	1.158 (0.702, 1.904)	1.182 (0.663, 2.109)
	Pneumonia+	1.042 (0.756, 1.420)	0.524 (0.207, 1.613)
Scaling	(2, 10]	1.00 (ref)	1.00 (ref)
	(0, 2]	0.902 (0.528, 1.539)	0.899 (0.411, 1.942)
	(10, 20]	1.239 (0.707, 2.185)	1.321 (0.704, 2.471)
	(50, 300]	1.012 (0.556, 1.862)	0.700 (0.328, 1.473)
